# Tracking everyday emotional experiences in university students with the distinct mood assessment questionnaire

**DOI:** 10.1007/s12144-025-08991-6

**Published:** 2026-02-27

**Authors:** Denise J. van der Mee, Lianne P. de Vries, Lydia Krabbendam

**Affiliations:** 1 Department of Clinical, Neuro, and Developmental Psychology, VU University Amsterdam, Van der Boechorststraat 7 Amsterdam, Amsterdam, 1081 BT The Netherlands; 2 Department of Biological Psychology, VU University Amsterdam, Amsterdam, The Netherlands

**Keywords:** Ecological momentary assessment, Mood, Distinct mood assessment, University students, Exam stress, Well-being

## Abstract

Ecological Momentary Assessment (EMA) has become a central method for studying emotional experiences in daily life. However, the need to keep EMA surveys brief to enhance participant engagement limits their ability to capture diverse emotional states. This exploratory study introduces the Distinct Mood Assessment (DMA), an alternative approach that measures a broad range of emotions. Participants select from 26 mood adjectives and rate their intensity, allowing for nuanced emotion tracking while minimizing survey length. Using a sample of 152 university students, we examined the feasibility of the DMA, exploring its acceptability, emotional variety, ability to capture distinct emotional states, and effectiveness in tracking emotional changes during a 28-day examination period. Results showed high compliance (72.8%), with participants completing the DMA in under 45 s, and reporting on all emotions across its full scale. However, the frequency with which an emotion was reported varied widely across participants. Therefore, emotions reported did not cluster reliably into broader constructs and individual emotions should be analyzed separately. The DMA proved effective in monitoring emotional responses to academic stress, highlighting both individual variability and common trends. These findings emphasize the importance of considering individual differences in emotional resilience and vulnerability, which could inform future interventions aimed at enhancing student well-being.

**Supplementary Information:**

The online version contains supplementary material available at 10.1007/s12144-025-08991-6.

## Introduction

Over the past two decades a world without Ecological Momentary Assessment (EMA) to study emotional experiences in daily life has become unimaginable to many researchers across various disciplines. This methodology allows researchers to not only study emotion dynamics when they occur, but also various related factors, including the social environment (e.g. Kesselring et al., 2021; Neff et al., 2021), the physical environment (e.g. Bergou et al., 2022; Kondo et al., 2020), emotion regulation styles (see meta-analyses by Boemo et al., 2022) autonomic nervous system activity (see review by Weber et al., 2022), physical activity (see review by Degroote et al., 2020), and psychopathology (Beames et al., 2021; Ben-Dor Cohen et al., 2023). To increase feasibility for and engagement of the participants in EMA studies, researchers feel the drive to make the EMA surveys as short as possible. Although this approach improves participant engagement, brief EMA surveys are limited in their ability to capture distinct emotional states. Here, we investigate an alternative approach to measure emotional experiences, namely. distinct mood assessment (DMA). DMA presents participants with a range of emotion adjectives, allowing them to select all that apply to their current mood. For each selected emotion, they rate its intensity. This method captures a broad range of mood states, but limits the burden for participants.

During a typical EMA study on emotional experiences, participants are requested to answer several short surveys per day over a period of several days. To obtain optimal adherence, researchers weigh the cost and benefits of gathering data at high density versus being able to measure over longer periods of time. A major consideration is the length of the EMA questionnaire itself. The questionnaire length influences the perceived burden and compliance of participants, as investigated by Eisele and colleagues in 2022. The authors compared the engagement and compliance of a 14-day EMA design that differed in questionnaire length (60 items versus 30 items) and prompt frequency (9 times a day versus 6 times a day). The longer questionnaire was considered more burdensome by the participants, but no effect was found for prompt frequency or an interaction effect between questionnaire length and prompt frequency. These results directly translated into a lower compliance for the longer questionnaire irrespective of prompt frequency.

To keep questionnaires as short as possible, researchers thus put effort in designing EMA surveys that only include the items absolutely required to measure the constructs of interest. In the field of emotion research, the Positive and Negative affect scale (PANAS) has been a popular tool, in which individuals rate their affective experience on a 1–7-point Likert scale. The original 20 items PANAS has been successfully translated to the 10 item PANAS short form (Thompson, 2007). Subsequent factor analyses show that the PANAS short form loads onto two factors: positive affect (items: determined, attentive, alert, inspired, active) and negative affect (items: nervous, upset, ashamed, hostile). The PANAS short is frequently used to assess emotion dynamics in daily life with EMA and items are usually averaged into positive and negative affect.

However, there are drawbacks to this approach. First, by using two categories based on valence, we only get a broad overview of the actual emotions experienced by individuals in their daily lives. For example, we might know that participants are experiencing higher or lower overall positive affect, but we do not know whether this is due to feeling more active or inspired. Additionally, despite different emotions share the same valence the emotions included in each factor vary substantially from one another. Feeling nervous is a vastly different emotional experience compared to feeling hostile. This severely limits the conclusions that can be drawn on actual emotional states and hampers comparison across multiple times points, since the same average score on negative affect on two time points could be due to completely different negative emotions. Second, the PANAS short consists predominantly of high arousal states. Low arousing positive feelings such as relaxed and content or low arousal negative feelings such as down are not included.

Keeping the number of assessed emotions at a minimum severely limits the complexity of the data on everyday life emotional experiences being collected, while measuring emotional experiences with high diversity in daily life is crucial within the field of mental health research. As a concrete example, emotional granularity refers to the ability of individuals to accurately distinguish between emotions of a similar valence and arousal state (such as anger and frustration, excitement, and enthusiasm, or happy and cheerful; Smidt & Suvak, 2015). Being able to distinguish positive emotions, i.e., a higher positive emotion granularity, has been associated with adaptive coping and successful self-regulation (Kashdan et al., 2015). The recent COVID-19 pandemic underscores the critical role of protective factors such as emotional granularity. During the pandemic young adults were most at risk for psychological distress (Glowacz & Schmits, 2020; Hamilton & Gross, 2021). Research showed that specifically pre-pandemic distress (Shanahan et al., 2022) and resilience (De Lorenzo et al., 2023) were a major driver of pandemic induced stress in this young adult population, highlighting the critical role of protective factors such as emotional granularity. Studying these factors in the context of potential future global threats is essential. However, the theory on emotional granularity is in its infancy (Tan et al., 2022). To obtain an accurate reflection of an individual’s emotional granularity, multiple emotions of the same valence and arousal state need to be included in a study. Likewise, within the promising field of digital phenotyping, having a good representation of emotional states is crucial. Digital phenotyping refers to the quantification of human behavior in daily life on a moment-by-moment basis with the use of personal digital devices (Onnela, 2021). A vast amount of objective data can be collected using an individual’s personal smartphone and associated wearables such as smart watches. The ultimate goal of such phenotyping is to predict subjective human behavior without the requirement of subjective human input. However, to create objective phenotypes, we first need to validate whether they relate to emotions of the same arousal and valence in a consistent manner, while distinctly differentiating from emotions of different arousal and/or valence states. This validation is challenging if emotions are measured using a limited set of items.

To diversify the number of emotions measured in daily life researchers developed new multi-item scales (see Fredrickson, 2013; Jones et al., 2024; Schmitt et al.,^2024^) or opt for an open-ended question, allowing individuals to freely describe their emotions. This latter method requires only a single question, in contrast to using numerous fixed questions. Given the potential to capture a similar richness of data, open-ended questions are an appealing approach in terms of reducing participant burden and improving adherence. For example, Nilsson et al. (2024) applied the open question approach to examine the association between language use and alcohol consumption, and Hoemann and colleagues explored the relationship between emotional granularity and cardiorespiratory physiological activity (2021) or emotional granularity and mental health (2024) using this approach. Although multi-item and open-ended approaches provide a more natural and diverse understanding of emotional experiences, it has a major limitation: participants may report a wide variety of emotions, leading to a large set of emotions with little repeated occurrence within or between individuals (Hoemann et al., 2021, 2024). Furthermore, the effects of these alternative designs on the average time it takes to answer a prompt, participant adherence, and perceived burden have not been studied. Additionally, the reported emotions still need preprocessing before analyses. Nilsson and colleagues (2024) used natural language processing to convert the reported emotions into a numerical array called word embedding, while the study by Hoemann and colleagues in 2021 asked participants to link their reported emotions to a list of 18 adjectives during an end of the day survey.

To our knowledge, no prior study has applied a method that merges the flexibility of open-ended emotion reporting with the structure of fixed-item scales in an in-the-moment EMA design. Building on the approaches of Fredrickson (2013) and Hoemann and colleagues (2021, 2024), we propose a new method for capturing a broad set of mood states in EMA studies: the distinct mood assessment (DMA). The DMA asks participants to select the emotions they are currently experiencing from a list of 26 adjectives. Participants are instructed to choose as many items as necessary to describe their current mood. For each selected emotion, they receive a follow up question to rate the respective item on a scale of 1 to 10. This hybrid format aims to combine the analytical strengths of standardized scales with the experiential relevance and participant autonomy of open-ended methods. In doing so, the DMA addresses long-standing challenges in capturing individual emotional granularity while maintaining comparability across time and participants. By combining this multiple-choice approach with follow-up questions, we aim to reduce the number of individual questions and questionnaire length, while still collecting information on a diverse range of mood states. Furthermore, this approach keeps the data structured, limiting preprocessing needs. While this method is not proposed as the definitive solution to capturing emotional experiences, it represents an exploratory attempt to optimize data collection across a diverse range of emotions with minimal participant burden. By addressing some of the limitations of existing methods, the DMA offers potential for a more complex assessment of everyday emotional experiences, though it also requires further investigation and refinement. Therefore, to explore the feasibility of this design, 152 university students participated in an EMA study during a 28-day period surrounding an examination. On each day participants received five prompts in which they were asked to fill in the DMA combined with questions regarding their surroundings, activities, and social environment.

The aim of this exploratory study is fourfold: (1) Quality of the DMA: We assess the acceptability of the DMA by measuring the subjective and objective burden. We explore whether participants feel the DMA accurately reflects their emotional experiences in daily life and the extent to which completing the DMA affects their subjective emotional experiences, (2) Frequency and Variety of Emotions: We explore how many emotions participants report per prompt, how frequently specific emotions are reported, and the individual differences in the variety of emotions selected, (3) Capturing Distinct Emotional States: We explore if the DMA can effectively capture distinct emotional states using exploratory factor analysis, and (4) Tracking Emotional Changes: we examine the performance of the DMA in tracking changes in emotions, particularly in response to and recovery from stressful events.

## Methods

### Participants

The research population consists of 152 students. Of this sample 105 participants were biological female, 46 were biological male and one preferred not to say. Of the biological female participants two identified as male and two as non-binary. Of the male participants one preferred not to say their gender identity. The person that did not wish to declare their biological sex identified as non-binary. This resulted in 101 cis-gender female, 45 cis-gender male, three non-binary, two transgender participants and one whom preferred not to declare their gender. Most participants were in the age range of 18–21 years old (*N* = 100), followed by 22–25 years old (*N* = 41), 26–30 years old (*N* = 8), and finally 30–55 years old (*N* = 3). Of the participants, 128 were bachelor students, of which 98 in their first year, and 24 were master students. There was a total of 61 international students who participated. Seven participants reported taking medication for mood disorders and eight participants for ADHD. More information on the study population can be found in the Project description on the Exam Stress Project OSF page (https://osf.io/rd9pm/).

The data was between October 2023 and February 2025. The inclusion criteria were: (1) they were students at the university with an exam week, (2) had an exam (reports and presentations were not counted), (3) mastered the English language, (4) were 18 years or older, (5) owned a smartphone, and lastly (6) were willing to download an application. There were no exclusion criteria. Participants could choose to be compensated 250 credits or 35 euros on a bol.com voucher. This study has been assessed by the Standing Committee on Science and Ethics (VCWE) of the Faculty of Behavioral and Movement Sciences, Vrije Universiteit Amsterdam and is in line with the ethical guidelines of the faculty (file: VCWE-2023-090). The privacy of the participants is protected by creating a study ID to which only the principal investigator and researchers had access.

### Study design

The total duration of this observational study was four weeks (28 days). During this period, participants were asked to complete five questionnaires per day about their emotions and activities. At the start of the study, students completed two questionnaires and at the end, a short questionnaire was administered about their experience during the study. The duration of four weeks was chosen to map distinct phases of academic life. Week one represents a typical teaching week, where students attend lectures and working groups as they normally do, with exam stress gradually rising. In week two, the exam is approaching, and many students will start actively studying for the exam. In week three, the exams take place. Finally, in week four the exams are over, with students returning to regular lectures and activities as a new teaching week begins Three periods were defined respectively to an individual’s examination schedule and start date. The exam anticipation starts at the starting day of the measurement and lasts until four days before the first examination. The examination period, in which students are expected to actively study for and partake in their exams, starts 4 days before their first exam and lasts until 5 h after the start of their last examination. The exam recovery period starts four hours after the start of their final exam and lasts until the final day of the study. Data collection took place between September 2023 and July 2024. This study is part of a larger study called the Exam Stress Project.

### Procedure

Recruitment was done through SONA application for participants. In addition, we distributed posters and flyers with information about the study in buildings of the Vrije Universiteit (VU). Data collection was performed in six periods during the academic year 2023/2024 and three periods during the academic year 2024/2025. The period aligned with the VU exam periods in October, December, January, March, May, and June. Upon registration, an information letter about the study was sent to the participant via email. When participants agreed to partake in the study an appointment at the laboratory of the university was scheduled. At the laboratory, the study was verbally explained and there was time to ask questions. Following, participants signed the informed consent and downloaded the EMA application on their smartphones.

The application m-path, developed by the KU Leuven, was used to collect Ecological Momentary Assessment (EMA) and daily diary data. M-Path follows the rules of the General Data Protection Regulation (GDPR) of the European Union. All data is stored, operated, and secured on servers of the KU Leuven. KU LEUVEN R&D provides all physical, electronic, and procedural safeguards for personal data. They apply measures such as identity management, pseudonymization and anonymization where possible, encryption where necessary, regular monitoring and evaluation of their security measures. This application was specifically chosen due to ease of use for researchers in setting up and scheduling questionnaires as well as monitoring the adherence.

An interval-contingent sampling scheme was chosen in which a prompt was sent within a fixed range of 60 min to decrease prompt anticipation. Each day five EMA prompts were sent that were expected to take ~ 2 min to answer (based on a pilot study). The participants were given 90 min for each prompt to complete the questionnaire. After 30 min, participants received a reminder. The time between two prompts had a possible range of 30 min to 330 min. There was no time out specifications other than the time window of 90 min passing by. Time windows were adjusted depending on the participant’s lifestyle. The option was provided to participants to freely include extra prompts. For example, if participants went to bed at a later time than usual, they could choose to enter an extra prompt.

At each EMA prompt individuals were asked questions about their current emotional experiences, and dominant posture, physical surroundings, and activities engaged in during the past 30 min. All questions were either binary (yes/no) or multiple choice with multiple answer options. Based on the selected answer, possible follow up questions followed. Due to this structure the number of questions and time it took to answer the questionnaire was highly variable, but based on data from period 1, 2 and 3 was on average 70.5 s. The questionnaires are also available in the m-path library under the name ES_EmotionTracking of the researcher “Exam Garmin” identifier “zrxb5”.

Adherence to the EMA was monitored by the researchers. When participants reached a threshold of 50% adherence, they were contacted by the researcher via text messaging to inquire their reason for their low adherence and motivate them to increase their adherence. There was no incentivization or other forms of motivation to increase participants’ engagement. After four weeks participants received their compensation, uninstalled the application from their phone, and filled in an evaluation questionnaire.

### Materials

#### Distinct mood assessment

The aim of the DMA design was to get a more detailed overview of the emotions and individual affective states without overwhelming the participants, by providing a mixture between the classical way in which emotions are assessed in EMA studies and the study design of Hoeman and colleagues (2021). At each diary entry participants were offered a multiple-choice question on which they could select as many emotions as they saw fit to describe their current emotional experience out of a list of 26 items. The emotion items were adjusted from the PANAS-X (Haney et al., 2023) aimed to measure the 13 constructs (Table 1). In addition, individuals were provided with an open-ended question in which they could indicate if they experienced another specific emotion that was not on the list.

**Table 1:** Selected items based on emotional states

Construct	Items 1	Item 2
Energy	energetic	active
Attentiveness	attentive	concentrating
Excitement	excited	enthusiastic
Interest	Interested	Motivated
Self-assurance	proud	confident
Joviality	happy	cheerful
Serenity	calm	relaxed
Fatigue	tired	sluggish
Apathy	bored	apathetic
Sadness	sad	down
Anxiety	anxious	worried
Hostility	angry	irritable
Stress	stressed	overwhelmed

For each emotion, participants selected in the multiple-choice question, a follow up question was given to rate the intensity of this emotion on a scale of 1 to 10. A scale of 1–10 to rate the emotional intensity is uncommon but, in our opinion, reflects a more naturalistic scale for human subjects. In the non-scientific community, where most of our participants reside, it is far more common to encounter questions on forums and surveys on a scale of 1 to 10. Due to the frequent exposure to this scale the vast majority of the western population has a more intuitive feeling to rate something on a 1 to 10 scale compared to a 1 to 7 scale. We can see examples of the functionality of such scales in widely varying populations in the pain scale used in hospitals (Begum & Hossain, 2019) or the assessment of occupational stress by occupational physicians (Lesage & Berjot, 2011). Research has shown that the validity of such visual analogue scales (VAS) is good (Begum & Hossain, 2019; Guyatt et al., 1987; Lesage & Berjot, 2011).

#### Evaluation questionnaire

At the end of the study, participants were asked to fill in an evaluation questionnaire in which they reflected on (1) How they experienced the amount of diary entries per day (Far too little/too little/adequate/too much/far too much), (2) whether they thought their reported emotions reflected their experienced emotions during the day (No/Somewhat/Yes), (3) whether answering the EMA prompts disrupted their daily lives (No/Yes), and (4) to what degree they thought filling in the diary questions influenced their perspective and/or experience of your emotions (Not at all/Somewhat/Moderate/Strong), followed by an open ended question in which they could describe how it influenced their behavior.

### Data analysis

#### Acceptability of the DMA

The percentage of all beeps that were filled in by the participants was calculated to determine overall adherence to the study. Following, we computed a daily adherence proportion for each individual on each measurement day and explore whether the adherence changes over time (day of study 1:28) or as a factor of week type (anticipation, exam, recovery) using multilevel modelling. In these models the proportion adherence per day is the level1 outcome and the day of study/week type the level 1 predictors, which are clustered within individuals. Random intercepts and random slopes were set to covary. Regarding the response time we calculated the average time it took for individuals to answer a prompt. Following we explore whether the response time changes over time using multilevel modelling. Both these models were run in R with the packages “lme4” and “lmerTest”.

To explore the subjective experience of participants with the DMA regarding burden and influence of the DMA on their emotional experience we will calculate the frequency of each answer category for the evaluation questionnaire items. The open-ended question regarding how the DMA influenced their emotions was manually recoded into separate variables. Based on the answers four variables could be identified: awareness, intensification, reflection, and regulation. Responses are compared using Chi-Square goodness-of-fit tests with Monte Carlo Simulation to account for frequencies below five.

#### Frequency and variety of emotions

To explore whether all emotions included in the DMA are used across their complete range and in what frequency, we counted the total number of times each emotion was selected across the complete study sample. Additionally, we computed the overall mean, standard deviation, minimum and maximum for each emotion. A list of the emotions that were reported in the open ended “other” question is given. In addition, we explored individual differences in emotion selection in three ways. First, we counted how many individuals selected an emotion at least once, at least five times, at least ten times, or at least twenty times during the entire study period. Second, for each individual we explored the number of emotions reported at each prompt (mean, SD, min, max). Third, we counted how many of the 26 emotions each individual selected at least once throughout the study.

#### Capturing distinct emotional States

The assess whether the items of the DMA load on the expected factor of Table 1 we performed confirmatory factor analyses (CFA) using the R package “lavaan” function cfa() with the estimator set to “MLR”. Given that participants in our design may not select certain items at all we are at risk of having variables with a standard deviation of zero. This will limit our ability to perform multilevel confirmatory factor analyses to examine the factor structures at different levels of data. Furthermore, multilevel CFA requires a theoretical model that defines how the factor structure varies across measurement levels.

Therefore, we first performed CFA on individual-centered values and test whether the same model fits the individual mean scaled values. For both models, we assessed discriminant validity to determine whether the factors are distinct from one another using the Fornell-Larcker criterion. This method compares the square root of the average variance extracted (AVE) for each construct with the correlations between constructs (Fornell & Lacker, 1981). Following, the composite reliability is assessed using a measure of the internal consistency. This is preferred over Cronbach’s alpha as it accounts for the different factor loadings of the indicators, providing a more accurate measure of internal consistency (Peterson & Kim, 2013). Composite reliability is calculated by taking the squaring the sum of the standardized factor loadings divided by the squaring the sum of the standardized factor loadings plus the sum of the error variances. The individual centered approach provides insight into the accuracy of the tool to detect between-individual reliability, while the individuals mean approach assesses within-individual reliability. Depending on the number of items and individuals with non-zero standard deviation, we evaluated if these findings can be replicated using multilevel factor analysis.

#### Tracking emotional changes

To assess how much variance in emotions was due differences between individuals we calculated the ICC on a model including solely clustering within subjects using the R package “nlme” function lme() and R package “performance” function icc(). To assess how much variance in emotion was due to the different periods surrounding an examination we calculated the ICC on a model including both the subject identifier and the type of week (anticipation/examination/recovery). The ICC for week type was calculated by subtracting the ICC for clustering within individuals from the ICC of clustering within individuals and week type. For each emotion construct, we explore how they change over time in anticipation of exam, during the examination week, and during the recovery using piecewise growth modelling as an extension to multilevel analyses using the R package “nlme” function lmer(). In this model, the time periods are included as random slopes. This enables us to identify different trajectories based on the distinct phases of the study.

#### Power

A priori power analyses were run following the Growth Curve Power Illustration by Brian T. Keller in the Cran R documentation (Keller, n.d.) using the R package “mlmpower.” For the current analyses, the parameters were set to: ICC = 0.10, within cluster fixed effect size expectation = 0.65, and effect size expectation of the random slope = 0.3. No parameters were included for between effect size expectations since we are only looking at the effect of the focal level 1 parameter time. The results of the power analyses with 1000 replication, a sample size of 90, number of repeated measures of 70, and *p*-value of 0.001 indicated excellent power to detect a fixed effect of time (1.00 ± 0.00), excellent power to identify random slopes (1.00 ± 0.00).

#### Statistical significance

Given the number of analyses conducted within this Exam Stress Project, a stringent significance threshold is necessary to mitigate the risk of inflated Type I errors due to multiple comparisons across studies within the same sample. To address this concern, a significance threshold of *p* <.001 is adopted for all statistical analyses. By adhering to *p* <.001, this study ensures that reported findings are robust and less likely to be artifacts of multiple testing. This approach enhances the reliability and reproducibility of the results, consistent with established statistical recommendations for studies involving numerous analyses.

## Results

### Acceptability of the DMA

**Fig. 1:**
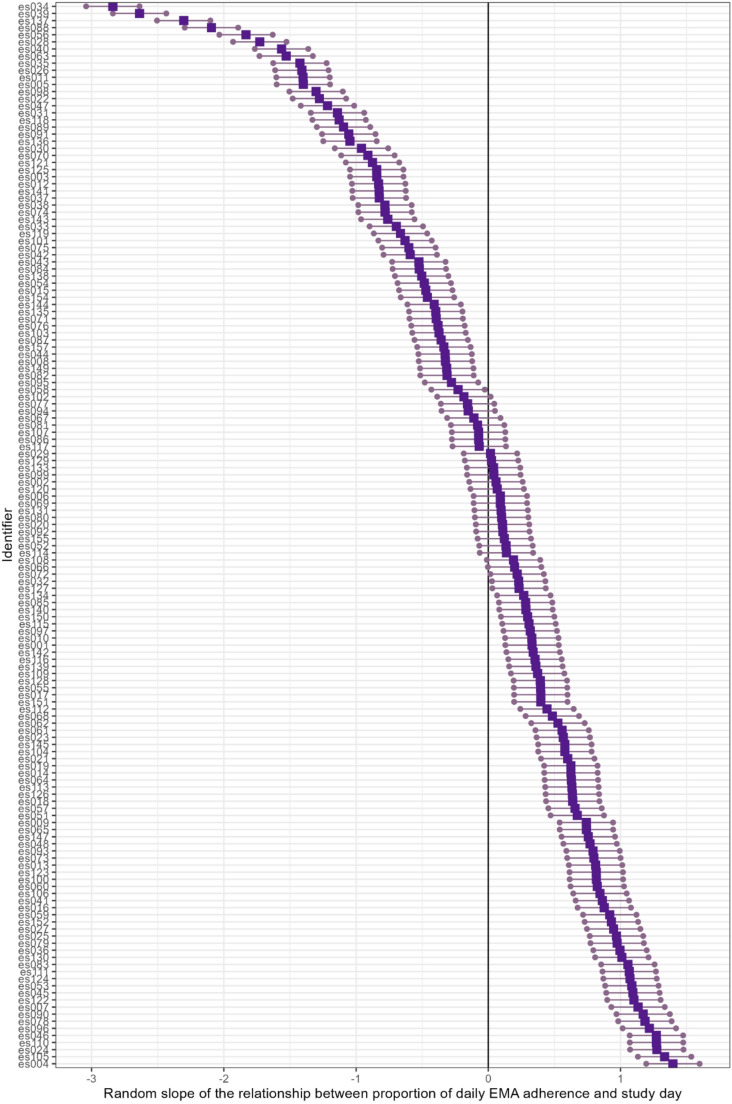
Individual differences between the change in daily adherence over time. *Note*. The strength of the individual’s random slope is represented by dark purple squares. Round purple dots connected by a purple line represent the 95% CI as based on the multilevel model. The solid black vertical line is placed at zero. Purple boxes below the zero line represent a decrease in daily adherence over time, while purple boxes above the zero line represent an increase in daily adherence over time. When the 95% CI includes the zero lines there is no change in daily adherence time. The results are plotted in order from smallest to largest individual random slopes values to aid interpretation

On average 106.69 prompts per participant were answered (range = 16–149, SD = 25.39), with a total number of 16,004 observations. The mean adherence to the EMA was 72.8% (range = 10.6–99.3, SD = 17.8). The mean percentage daily adherence was 72.7% (range = 10.6–100.0, SD = 99.4). The percentage of daily adherence showed a slight decrease over time (β = −1.10, SE = 0.08, t = −13.00, *p* <.001), but was variable between participants (Fig. 1). There was no difference in percentage daily adherence based on the type of week the data was collected in (anticipation/examination/recovery). On average, it took participants 44.08 s (range = 10–299, SD = 34.56) to answer the prompts. Over the course of the study the time it took to answer decreased (β = −8.27, SE = 0.40, t = −20.17 *p* <.001). which was variable across participants (slope SD = 3.96).

Data on the evaluation of the study was available for 148 participants. Of these, 50.7% indicated that the number of diaries they answered provided a good representation of their emotions during the day, 47.3% reported that it somewhat reflected their emotions, and 2.0% stated that it did not reflect their emotions (χ²(2) = 65.53, *p* <.001). Less than a quarter of the individuals (23.0%) indicated that the EMA disrupted their daily life (χ²(1) = 43.24, *p* <.001). Regarding prompt frequency, no participants indicated that there were far too little, 3.4% indicated it was too little, 72.3% indicated it was adequate, 22.3% indicated it was too much, and 2.0% indicated it was far too much (χ²(4) = 276.73, *p* <.001). Of the participants 79.1% indicated that the EMA influences the experience of their emotions to some degree (52.0% somewhat, 25.7% moderate, 1.4% strong; χ²(3) = 77.35, *p* <.001).

It was reported by 48% (χ²(1) = 0.24, *p* =.68) that the reporting of their emotions influenced their awareness of the emotions and by 9.5% (χ²(1) = 97.30, *p* <.001) that it intensified their emotions. Only a minority of individuals reported increased reflection on their emotional state (17.6%; χ²(1) = 62.27, *p* <.001) or that they actively engaged in emotion regulation to change their negative emotions (12.2%; χ²(1) = 83.82, *p* <.001).

### Frequency and variety of emotions

Table 2 gives an overview of how often each emotion is reported as a proportion of the whole sample and their respective distribution. Each emotion is reported over its full range in the study at large, but some emotions are reported substantially less than others. Based on the full dataset participants selected between 0 and 16 emotions per entry, with an average of 2.39 (SD = 1.49), in the case of 0 participant selected a free entry emotion. The mean number of reported emotions per prompt varied between individuals ranging from 0.98 to 8.91, with 38.0% of the participants having a mean number of reported items of below two, 36.7% having a mean between two and three, 25.3% having a mean larger than three.

**Table 2:** Frequency & distribution of items

Item	*N*	%	M	SD	Min	Max
Angry	188	1.2	0.09	0.82	0	10
Apathetic	441	2.8	0.20	1.20	0	10
Sad	539	3.4	0.24	1.33	0	10
Bored	678	4.2	0.29	1.43	0	10
Interested	720	4.5	0.34	1.60	0	10
Irritable	742	4.6	0.32	1.52	0	10
Confident	751	4.7	0.37	1.68	0	10
Enthusiastic	779	4.9	0.37	1.67	0	10
Proud	806	5.0	0.40	1.76	0	10
Down	911	5.7	0.39	1.65	0	10
Overwhelmed	911	5.7	0.44	1.82	0	10
Attentive	1002	6.3	0.47	1.84	0	10
Excited	1112	6.9	0.52	1.95	0	10
Cheerful	1162	7.3	0.55	1.99	0	10
Worried	1274	8.0	0.56	1.98	0	10
Energetic	1359	8.5	0.64	2.14	0	10
Sluggish	1413	8.8	0.65	2.15	0	10
Anxious	1461	9.1	0.62	2.03	0	10
Motivated	1642	10.3	0.76	2.30	0	10
Stressed	1866	11.7	0.81	2.34	0	10
Concentrated	1961	12.3	0.85	2.34	0	10
Active	2094	13.1	0.98	2.56	0	10
Relaxed	2594	16.2	1.19	2.77	0	10
Happy	2919	18.2	1.4o	3.02	0	10
Calm	3219	20.1	1.45	2.94	0	10
Tired	5803	36.3	2.62	3.61	0	10

**Fig. 2:**
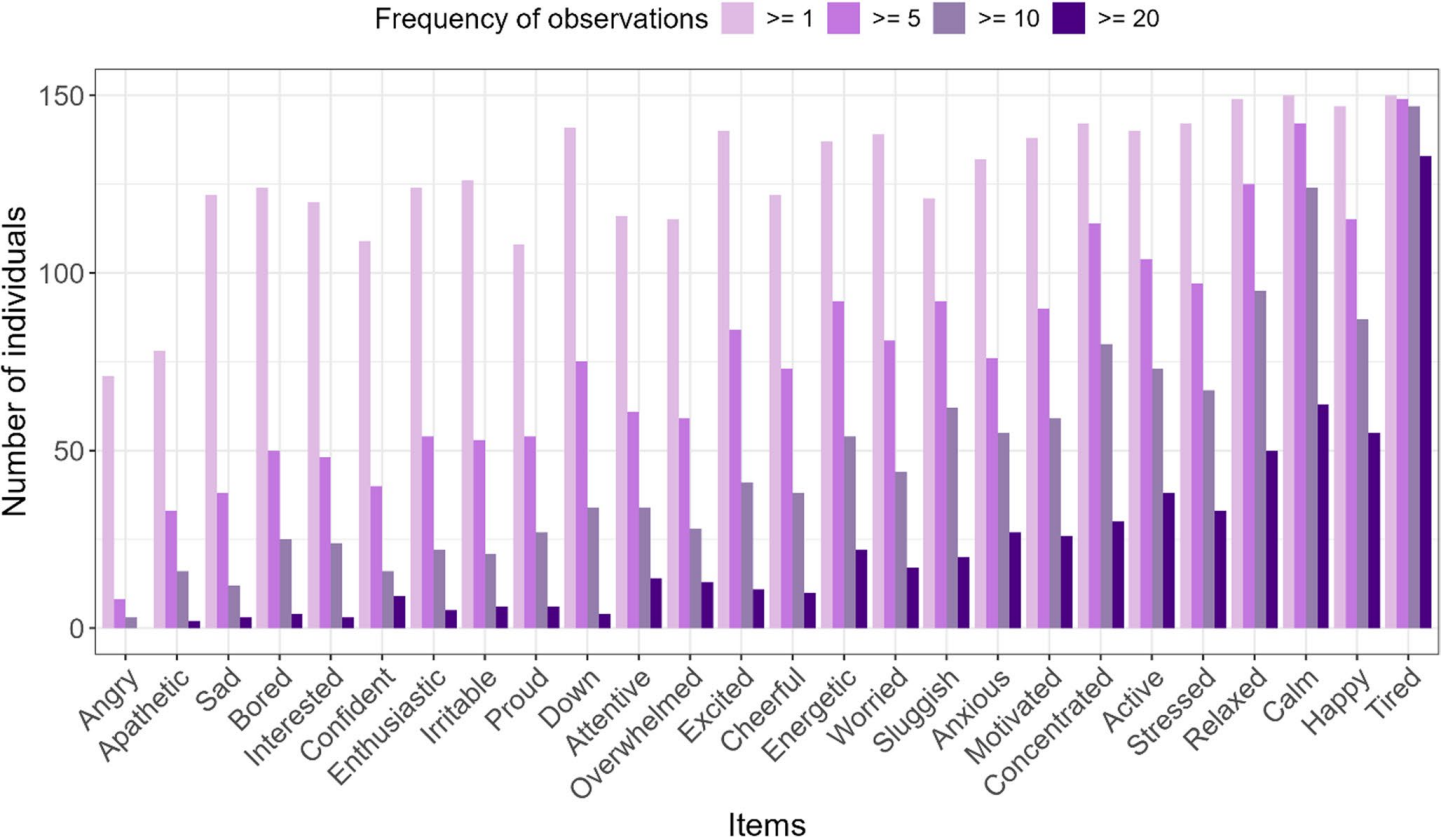
The number of individuals that reported on the item at different observation frequencies

Figure 2 shows the frequency with which individuals selected different items at least once, five times, ten times, or twenty times in the order of least to most frequently selected items. Of the 26 emotions, 24 were reported at least once by 70% of the participants and 14 at least five times by 50% of the participants. Strikingly, of all emotions *tired* is by far the most reported emotion. In almost 90% of all the participants this emotion was reported at least twenty times during the course of four weeks. The items *calm*, *relaxed* and *happy* are also reported very frequently. The items *angry* and *apathetic* were not reported at all by around half of the participants, and those who did select them did so only occasionally.

The number of items selected during the study ranged from 13 to 26 with a mean of 22.02 (SD = 2.93). Only 13 individuals selected each item at least once, and 30 reported less than 20 items. The items that were never reported by individuals were very variable. The open-ended question to provide additional emotions was used by 79 participants with a total of 308 entries and included the following emotions: (A) high arousal negative emotions: *agitated (n = 2), annoyed (n = 2), anticipating (n = 3), embarrassed (n =2), frustrated (n = 4), guilt (n = 12), hurried (n = 2), jittery/restless (n = 7), impatient (n = 1), nervous (n = 29), pissed (n = 1), relieved (n = 12), scared (n = 1); B) low arousal negative emotions: confused (n = 11), disappointed (n = 12), lonely (n = 4), melancholy (n = 2), numb (n = 6), resigned (n = 3), vulnerable (n = 1); C) bodily sensations: cold (n = 4), hot (n = 2), uncomfortable (n = 2), pain (n = 4), sick (n = 58), hunger (n = 16), drunk (n = 1), high (n = 2), horny (n = 2); lazy (n = 2), exhausted (n = 2); D) positive emotions: content (n = 34), curious (n = 1), grateful (n = 2), hopeful (n = 1), nostalgic (n = 1), optimistic (n = 1), patient (n = 1), thankful (n = 1), silly (n = 2); and E) motivation related: distracted (n = 7), focus (n = 4), pensive (n = 3), productive (n = 2), unmotivated (n = 14)*.

### Capturing distinct emotional states

Pairwise chi-square comparisons across the full dataset showed that overall, the emotions relate as expected within their valence and arousal quadrants (Supplementary Fig. 1). However, the CFA model did not converge. The high overall reporting of the item *tired* relative to their counterpart *sluggish* and the exceptionally low reporting of the items *bored* and *apathetic* caused problems. Using stepwise deletion of these factors indeed led to convergence of the model. The overall model fit on the remaining 11 emotion constructs (Table 3) indicated an acceptable to good fit (CFI = 0.95, TLI = 0.93, robust RMSEA = 0.018 [0.017–0.019], SRMR = 0.016). The discriminant validity analyses indicated that factors for stress and anxiety (*r* =.69), interest and attentiveness (*r* =.57), interest and self-assurance (*r* =.59), and excitement and joviality (*r* =.56) showed no discriminant validity with large correlations between factors, indicating that they measure overlapping constructs. The composite factor reliability was poor, ranging between 0.26 and 0.47 (Table 3).

**Table 3:** Factor loadings and composite factor reliability of scaled individual centered values

Factor	Construct	Item	Loading	*R*^2^ unexp.	Reliability
1	Anxiety	Worried	0.49	0.76	0.41
		Anxious	0.53	0.72	
2	Serenity	Relaxed	0.48	0.77	0.36
		Calm	0.46	0.79	
3	Interest	Interested	0.31	0.90	0.26
		Motivated	0.46	0.79	
4	Excitement	Enthusiastic	0.52	0.73	0.41
		Excited	0.51	0.74	
5	Sadness	Down	0.60	0.64	0.47
		Sad	0.50	0.75	
6	Attentiveness	Concentrated	0.57	0.67	0.36
		Attentive	0.36	0.87	
7	Energy	Energetic	0.60	0.63	0.45
		Active	0.47	0.78	
8	Hostility	Irritable	0.56	0.69	0.40
		Angry	0.44	0.81	
9	Joviality	Happy	0.48	0.77	0.31
		Cheerful	0.38	0.86	
10	Stress	Stressed	0.56	0.68	0.39
		Overwhelmed	0.42	0.82	
11	Self-assurance	Proud	0.40	0.84	0.32
		Confident	0.48	0.77	

The CFA model fit on the mean values did not converge as well. After inspection the correlation between the factors multicollinearity was identified and factors were merged (factor 1 = sluggish & tired; factor 2 = anxious, overwhelmed, stressed & worried; factor 3 = calm & relaxed; factor 4 = attentive, cheerful, concentrated, confident, enthusiastic, excited, happy, interested, motivated & proud; factor 5 = apathetic & bored; factor 6 = down & sad; factor 7 = active & energetic; factor 8 = angry & irritable). The resulting model of seven factors converged but indicated an insufficient fit of the data (CFI = 0.84, TLI = 0.81, robust RMSEA = 0.086 [0.077–0.097], SRMR = 0.080). Therefore, no further analyses were performed on this level. Due to high variance in the items not reported between individuals an insufficient sample size was present to perform multilevel factor analyses.

### Tracking emotional changes

**Fig. 3:**
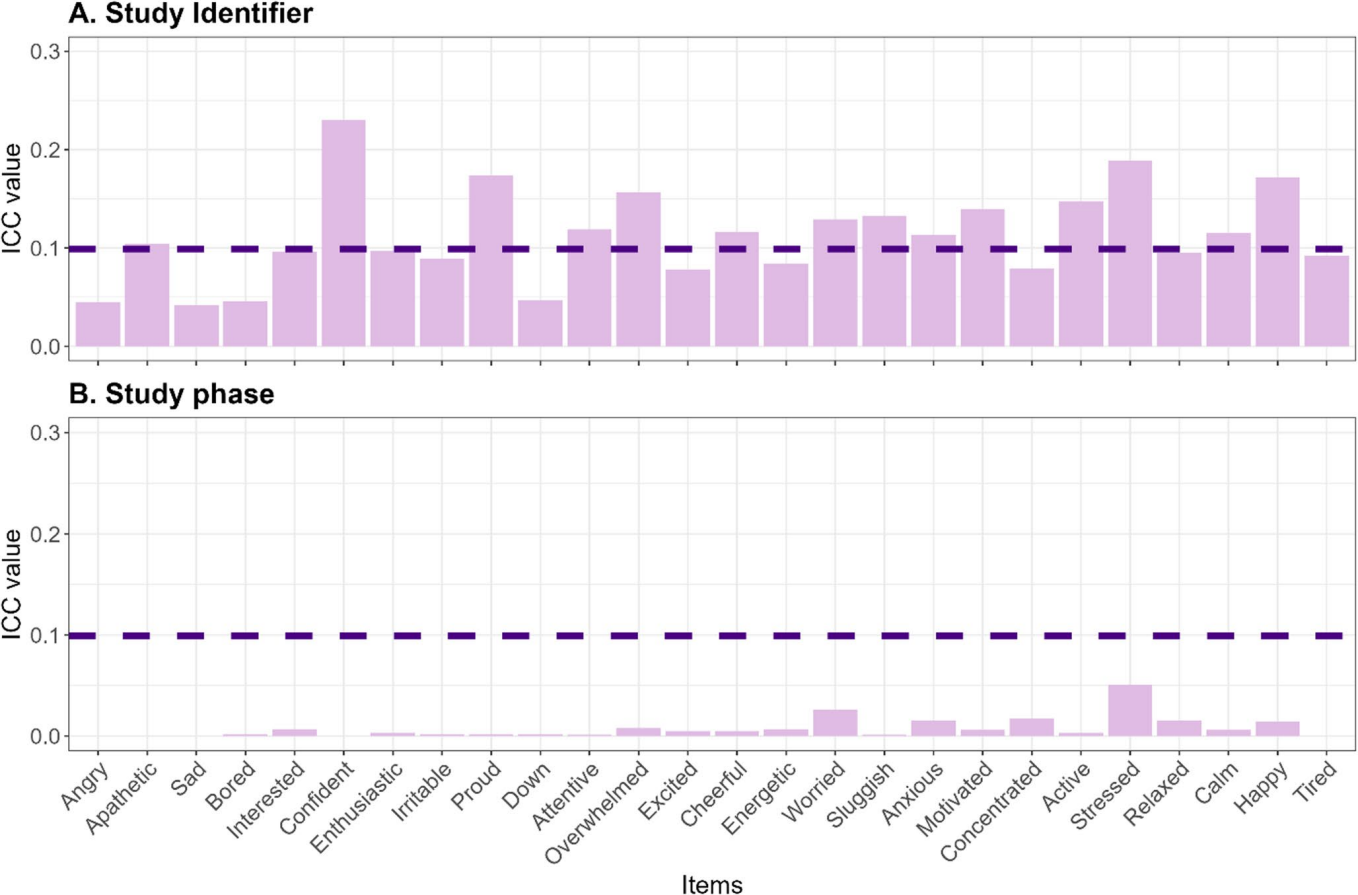
Intra-class correlation in items with study identifier or study phase as grouping factor

We perform the analyses on emotional changes on the individual item levels, because the CFA showed no reliable factors. The ICCs for clustering within individuals ranged from low to medium (Fig. 3 A). For several items (*active*,* anxious*, *apathetic*, *attentive*, *calm*, *cheerful*, *happy*, *motivated*, *overwhelmed*, *proud*, *sluggish*, *stressed*, *worried)*, more than 10% of the total variance in the outcome can be attributed to differences between individuals, validating the multilevel approach. Clustering within study period showed low ICC (Fig. 3B), indicating that we do not need to account for clustering within these periods.

The piecewise growth models shows that 13 emotions changed, on average across all participants, during at least one of the periods (Table 4). During the anticipation period the positive emotions *excited*,* calm*, and *relaxed* decreased, while the negative emotion *stress* increased. During the examination, the positive emotions *excited*,* calm*, and *relaxed* continued to decrease. In addition, the positive emotions *active*,* energetic*,* enthusiastic*,* happy*, and *interested* also decreased. The negative emotions *stress* continued to increase, in addition the negative emotions *anxious*, and *worried* also increased. During the recovery period, the positive emotions *energetic*,* enthusiastic*,* excited*, and *interested* continued to decrease. In addition, the positive emotions *concentrated* and *motivated* decreased. The emotions *angry*,* apathetic*,* attentive*,* bored*,* cheerful*,* confident*,* down*,* irritable*,* overwhelmed*,* proud*,* sad*,* sluggish*, and *tired* showed not significant change, though the emotions *confident*,* proud*, and *sad* showed trends.

**Table 4:** Piecewise growth models: change of emotions over time

		Anticipation			Examination			Recovery		
Item	Intercept	Est.	SE	*p*	Est.	SE	*p*	Est.	SE	*p*
Angry	0.088	0.001	0.001	0.31	0.000	0.001	0.84	−0.001	0.001	0.36
Apathetic	0.193	0.002	0.001	0.068	−0.001	0.002	0.83	0.000	0.001	0.81
Sad	0.204	0.003	0.001	0.060	0.007	0.002	**0.004**	0.000	0.001	0.86
Bored	0.344	−0.001	0.001	0.23	−0.005	0.002	0.034	−0.003	0.001	0.009
Interested	0.464	−0.002	0.001	0.015	−0.016	0.002	**< 0.001**	−0.007	0.001	**< 0.001**
Confident	0.378	0.000	0.001	0.94	−0.001	0.003	0.70	−0.003	0.001	***0.004***
Enthusiastic	0.469	−0.003	0.001	***0.003***	−0.012	0.003	**< 0.001**	−0.004	0.001	**0.001**
Irritable	0.363	0.001	0.001	0.50	0.000	0.002	0.90	−0.003	0.001	0.008
Proud	0.386	−0.001	0.001	0.32	0.008	0.003	***0.002***	−0.002	0.001	0.136
Down	0.375	0.003	0.001	0.033	0.004	0.003	0.15	−0.002	0.001	0.033
Attentive	0.457	0.003	0.002	0.086	−0.002	0.003	0.33	−0.002	0.002	0.27
Overwhelmed	0.411	0.005	0.002	0.023	0.012	0.005	0.007	−0.005	0.003	0.071
Excited	0.666	−0.004	0.001	**0.001**	−0.018	0.003	**< 0.001**	−0.005	0.001	**0.001**
Cheerful	0.57	−0.004	0.002	0.014	−0.009	0.003	0.007	0.003	0.002	0.18
Worried	0.456	0.004	0.002	0.010	0.036	0.005	**< 0.001**	−0.005	0.002	0.016
Energetic	0.756	0.001	0.001	0.53	−0.016	0.003	**< 0.001**	−0.009	0.002	**< 0.001**
Sluggish	0.718	0.002	0.002	0.21	−0.004	0.004	0.21	−0.003	0.002	0.14
Anxious	0.522	0.004	0.002	0.038	0.030	0.005	**< 0.001**	−0.004	0.002	0.077
Motivated	0.772	0.002	0.002	0.16	0.003	0.004	0.39	−0.007	0.002	**< 0.001**
Concentrated	0.827	0.003	0.002	0.038	0.013	0.005	0.009	−0.009	0.002	**< 0.001**
Active	1.113	−0.001	0.002	0.73	−0.017	0.004	**< 0.001**	−0.007	0.002	***0.002***
Stressed	0.594	0.011	0.002	**< 0.001**	0.063	0.008	**< 0.001**	−0.008	0.003	***0.004***
Relaxed	1.471	−0.011	0.002	**< 0.001**	−0.039	0.004	**< 0.001**	−0.008	0.003	0.008
Calm	1.657	−0.007	0.002	**< 0.001**	−0.029	0.004	**< 0.001**	−0.006	0.004	0.15
Happy	1.511	−0.007	0.002	***0.004***	−0.033	0.004	**< 0.001**	0.003	0.004	0.42
Tired	2.756	−0.003	0.002	0.19	−0.009	0.006	0.14	−0.007	0.003	0.031

Significant effects are depicted in bold; trends are depicted in italic bold

**Fig. 4:**
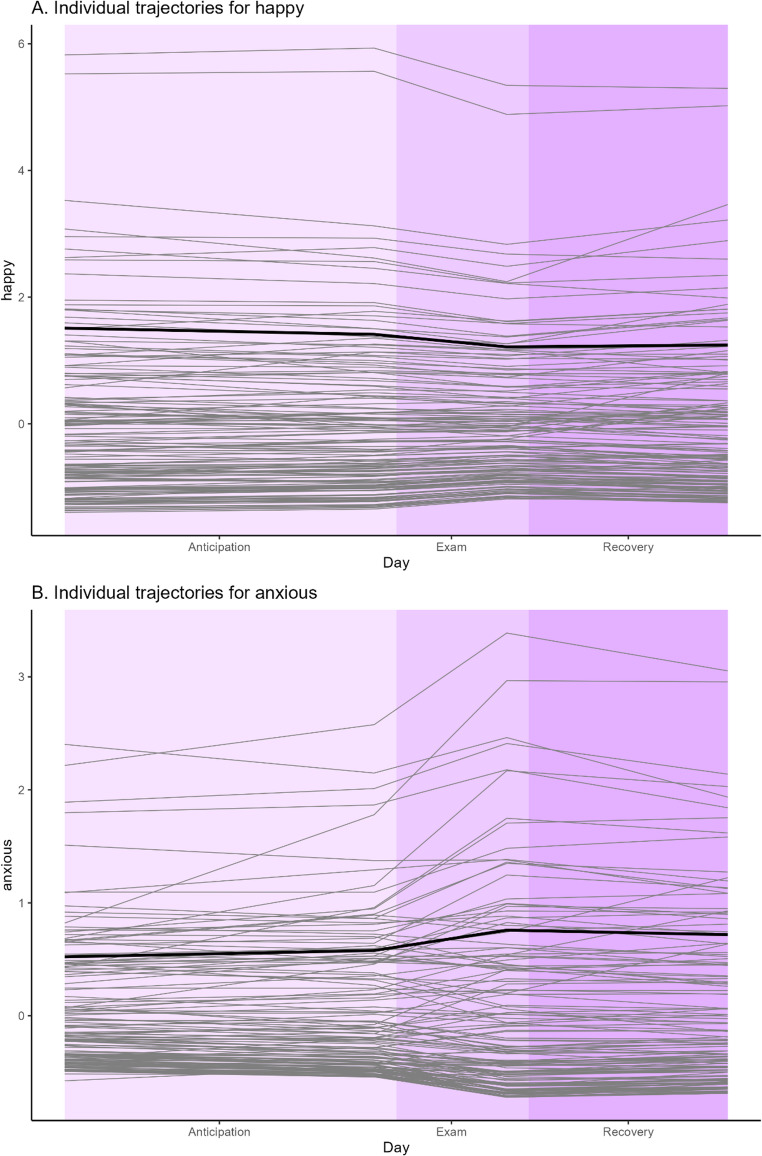
Individual differences in the change of happy and anxious over the examination phases

In all models there were individual differences in the strength and direction of the slopes. Examples are given for the change in *anxious* over time (Fig. 4 A) and the change in *happy* over time (Fig. 4B). Regarding *happy*, the differences in slopes between individuals are modest. For *anxious*, however, more substantial differences can be observed. Especially during the examination week there appears to be a group of individuals whose anxiety rapidly increases. This difference in the extent of variability is also observed in the other emotions (see Appendix 1). Like happiness, there are modest differences between individuals for the emotions *attentive*,* active*,* calm*,* confident*,* energetic*,* enthusiastic*,* excited*,* interested*,* irritable*,* proud*, and *relaxed*. Like anxiety, there are more substantial between individual differences for the emotions *bored*,* cheerful*,* concentrated*,* down*,* motivated*,* overwhelmed*,* sluggish*,* stressed*,* tired*,* sad*, and *worried*. For the emotions *angry* and *apathetic* there were only a few individuals that differed from the others which is consistent with frequent reporting of these emotions by only a subsample of the population.

## Discussion

Applying EMA to study emotion dynamics in daily life has proven to be a valuable tool. However, the drive to keep the burden as low as possible has made EMA surveys on emotions too short to study individual differences in valence and arousal dimensions or emotional intelligence, such as emotional granularity. In the current study we explore a new tool, dynamic mood assessment (DMA), to get more diverse emotional experiences while keeping the burden within reasonable limits. A sample of 152 students at a Dutch University completed the DMA five times a day over a period of 28 days, which surrounded an examination. We show that the DMA is an innovative and feasible tool for studying emotional dynamics in daily life. Furthermore, the DMA has the potential to uncover unique patterns of change in emotions in response to real life stressful events. However, the inability to use standard.

data reduction methods such as factor analyses underscores unique issues that need to be explored further.

### Acceptability of the DMA

Overall, the acceptability of the DMA was good. The compliance to the DMA was on average 72.8% among the participants. This falls within the range expected for this population of 51.6% and 92.0% (M = 74.0, SD = 11.5) when using more traditional EMA surveys (van Roekel et al., 2019). In addition, only around 20% of the individuals reported that they perceived the number of prompts per day as too much. Together these findings suggest that providing individuals with a more dense array of emotions to select from does not increase experienced burden. Rather, this indicates that the burden of the DMA does not differ from traditional methods even with a prompting schedule of 5 times a day over a period of 28 days. An explanation for the positive evaluation of the DMA is the perceived control of participants over the EMA items. Instead of traditional EMA research where all participants follow the same protocol, the DMA allows participants to pick the emotions that are personally relevant. According to the self-determination theory, the experience of perceived control and autonomy are positively related to intrinsic motivation (Bureau et al., 2022; Reeve et al., 2008). Therefore, the increased autonomy when filling in the DMA could increase the motivation of participants to complete more surveys.

On average, participants completed the survey in around 45 s, which is less than initially expected. Furthermore, the completion time decreased over time, suggesting that as participants gained experience, navigating, and selecting emotions became easier. Consistent with previous studies, the adherence of participants on average lowered over time (Eisele et al., 2022; Wrzus & Neubauer, 2023). As already shown Eisele and colleagues (2022) decreased adherence over time was not related to the duration of the study. We find further support of this initial drop of adherence followed by a stabilization over time by our observation that the decrease became less pronounced over the course of the study. Surprisingly, there was no apparent drop in adherence during the examination week. Given that during that week students are actively studying for their exams, it would have been logical if adherence had dropped. One explanation could be that we had a highly motivated study population that strongly believed in the relevance of this topic, which could have aided the adherence even during more stressful times. Furthermore, there were considerable individual differences. An equal proportion of individuals showed an increase in adherence over time, rather than a decrease. Though a meta-analyses by Wruz and Neubauer investigated the influence if factors such as age, gender, and health status, none of the factors could explain differences in adherence. It would be interesting for future research to compare these groups of people based on personality characteristics or reported effect of the EMA on their emotional experiences. This could greatly aid strategies aimed at achieving the best possible adherence.

Consistent with previous research, most participants reported that filling in the DMA made them more aware of their emotional state (van Os et al., 2017). This could raise concerns that engaging in an EMA study focused on emotions may inadvertently function as an intervention (van Os et al., 2017). However, in this study, less than 20% of individuals reported that the DMA led to reflection on their emotional state or active emotion regulation. This suggests that while participants may become more aware of their emotions, most do not (conscientiously) engage in intervention-like behaviors. Further support for this idea comes from a study by De Vuyst et al. (2019), which found that conducting 10 EMAs of emotions per day over a period of 7 days did not affect participants’ emotional experience over time. On the other hand, Ludwigs et al. (2018) showed that paying increased attention to well-being for two weeks had a small positive effect on well-being in university students. Given these mixed findings, it would be valuable to further explore the effect of engaging in EMA on emotion regulation. In this study, the sample of individuals reporting emotion reflection or regulation effects was rather small, therefore we could not investigate whether in these individuals the experience of negative emotions indeed changed over the course of the experiment or whether these individuals differed from the others in their overall reporting of negative emotions. Moreover, due to the design of our study we expected motions to change in response to the naturalistic stressor further complicating such analyses. It is therefore highly recommended for future researchers to follow our approach and include such a short evaluation questionnaire at the end of their EMA study to further investigate this possible intervention effect.

### Frequency and Variety of Emotions & Capturing Distinct Emotional States

With the DMA, we were able to detect distinct emotional states; however, the selection of emotions included in the assessment could benefit from further fine-tuning. Alle emotions were used in its full range indicating that individuals select an emotion even if they experience them to only a modest degree. Furthermore, of the participants more than 70% reported on at least 24 out of 26 available emotions. Moreover, the frequency with which an emotion was reported varied widely across participants. There was no clear set of emotions that was reported on frequently by most participants. These findings highlight that daily life emotional experiences are highly individualistic. Our findings on the bandwidth of the human emotional experience is consistent with the findings of Hoeman and colleagues (2021, 2024). Applying a free labelling approach resulted in 429 unique labels across 67 adults during a 14 day study. Importantly, the most frequently reported items showed little with frequently used shorter PANAS scales such as the 20-item PANAS-scale (Watson & Tellegen, 1988), let alone even shorter tools. This is consistent with reported modest reflection of the DMA regarding the actual experienced emotions. Despite providing individuals with 26 emotional states based on a validated research tool, the PANAS-X (Haney et al., 2023), most of the individuals still did not think all the emotions they were experiencing during the day were available as an option. Interestingly, although participants were given the opportunity to add custom emotions, this option was rarely used. This could indicate that while some emotional states were perceived as missing, they were not salient enough to prompt active reporting—or that participants may default to provided labels for convenience, even if they’re not a perfect fit. Given the good compliance and acceptable burden of the DMA, further iterations of this tool provide a promising avenue to optimize personalized emotion tracking. For example, a hybrid approach could be applied which involves initially allowing participants to provide the emotions they most frequently experience, which would then form the basis of their personalized item set—with the option to adjust or add to these selections as the study progresses.

On average, individuals reported 2–3 emotions per entry, which is notably higher than the number of emotions reported in the free-label entry study by Hoemann and colleagues (2024), where participants selected an average of 1.51 emotions per entry in Study 1 (SD = 0.55) and 1.62 in Study 2 (SD = 0.73). This suggests that the hybrid design of the DMA may encourage richer emotional reporting compared to fully open-ended methods. Interestingly, the emotions reported at an entry did not belong to the same construct as indicated by the poor composite factor reliability. This inability to construct reliable factors suggests that the emotions captured in the DMA should be analyzed as individual items rather than as composite scores based on sums or means. Using single is not necessarily problematic, a recent study by Song and colleagues (2023) demonstrated that single-item measures have comparable predictive validity to multi-item mean scores in EMA research. Nevertheless, these findings suggests that EMA design has a considerable influence on the results. With the current design we could not replicate earlier findings on the good reliability of the PANAS-X (Haney et al., 2023). This contrast highlights how methodological design—in particular, user-driven selection versus full-scale rating—can substantially impact reliability outcomes. Whereas Haney et al. (2023) report robust factor consistency under forced-choice conditions, our more flexible design revealed emotional patterns that elude standard dimensions. One explanation can be that the DMA is more inviting to reflect on the emotional state thereby inducing higher emotional granularity. Another explanation is that this design reduces the likelihood of careless responding. Careless responding means that individuals respond to the EMA survey items without paying sufficient attention to the questions (Meade & Craig, 2012). In traditional designs, where individuals must rate every emotion, participants might reflect only briefly on their emotional state, leading them to quickly assign similar ratings to related emotions (e.g., rating both “happy” and “cheerful” based on a single general feeling of cheerfulness). By contrast, the necessity to choose specific emotions may prevent this kind of hasty responding. That careless responding does not happen in the DMA is supported by our data. With the DMA individuals did not simply pick the top emotion or only one emotion at the time to be done with the survey quickly, they consistently reported multiple emotions, indicating thoughtful engagement. Future research could benefit from qualitatively comparing participant experiences across traditional, open-ended, and hybrid methods to better understand how each format shapes emotional insight, reporting behavior, and perceived burden. Such insights could guide the development of more effective and user-centered EMA tools.

### Tracking Emotional Changes

The DMA proved to be a valuable tool to track changes in emotions in response to an academic exam. The changes in emotions in response to the different examination phases was largely as expected. On average across all participants, many positive emotions decreased in anticipation of an examination or during the examination period, whereas negative emotions increased. After the examination had passed specific emotions expressed during studying such as motivation and concentration decreased, while negative emotions no longer show a change over time. These results align with prior research indicating that students commonly perceive exams as significant stressors (Borghi et al., 2021; van Dopmeijer et al., 2021; Murphy et al., 2010; Spangler et al., 2002; Weekes et al., 2006). At the same time, our data revealed notable variability in emotional responses between individuals, echoing the work of Reeve et al. (2014). In their study, students completed emotion assessments both before and after an exam, revealing that while many reported negative emotions such as anxiety, shame, or anger, others expressed more positive feelings like pride and joy in anticipation of the test. Overall, the observed pattern of emotional changes in anticipation of an examinations is also consistent with the viewpoint of mental health as not only the absence of mental illness or only the presence of well-being (Keyes & Michalec, 2010). Both positive and negative emotions are influenced by this period and should be investigated both.

However, there were some unexpected findings. Positive emotions further decreased after the examination has passed, indicating that there seems to be a recovery of negative but not positive emotions in this time period. This suggests that the recovery of positive emotions might take longer. In addition, not all emotions showed a response to an examination at the population level. For some emotions, this could be explained by the rather low frequency at which these emotions were reported, such as angry and apathetic. For other emotions, it is less clear. For example, the emotion happy showed a stronger decreased in anticipation of an examination than the emotion cheerful. Likewise, there was a trend for an increase in feeling sad but not down. One potential explanation for this finding could be related to the cognitive processing of emotions in stressful periods. According to Davis et al. (2004), during peaceful and predictable circumstances, individuals have the cognitive capacity to consider various aspects of their emotions. However, in times of stress or uncertainty—such as the lead-up to an examination—this separation of emotions can become more cognitively demanding. The need to process affective stimuli quickly may outweigh the ability to evaluate them thoughtfully and deliberately. This theory has mostly been assessed in the relation between positive and negative affect (Dejonckheere et al., 2021) but could potentially explain why certain closely related emotions (e.g., *happy*, and *cheerful*) are processed differently under stress.

These findings underscore the added value of the DMA in examining emotional experiences during high-stress periods like examinations. Its ability to capture subtle distinctions between closely related emotions—such as “happy” versus “cheerful” or “sad” versus “down”—is particularly important when stress may alter how emotions are cognitively processed. Unlike traditional tools that aggregate responses into broad positive or negative affect scores, the DMA allows for a more fine-grained, item-level analysis that can detect nuanced shifts in emotional tone. This granularity provides insight into both the general and individual-level variability in emotional recovery patterns, offering a promising avenue for identifying students who are more vulnerable or resilient. As such, the DMA is especially well-suited to future research aiming to disentangle complex emotional dynamics over time and across diverse student profiles.

### Limitations

Despite the practical advantages of the DMA for studying individual differences in emotion trajectories or emotional intelligence, there are several important limitations that must be acknowledged. One major limitation is psychometric in nature. Specifically, attempts to perform a confirmatory factor analysis (CFA) on both the full and mean-level datasets failed to converge, indicating that the factor structure of the DMA is not robust. Although a reduced-item model showed acceptable fit indices, this model lacked discriminant validity, and composite reliability across factors was poor. These issues severely limit the interpretability of any factor-based conclusions and indicate that caution is warranted when deriving composite scores. We fully acknowledge that this represents poor fit. However, these psychometric characteristics may reflect the inherent design of the DMA, which captures dynamic, rarely co-occurring emotional experiences at the item level. Consequently, conventional CFA and traditional aggregation methods may not be well-suited for this type of data, and the observed limitations may instead point to the need for analytic approaches tailored to dynamic, person-specific affective measures. In addition, because DMA items are rarely reported simultaneously, it is not possible to calculate sum or average scores for items within the same affective cluster, further restricting traditional analyses and precluding direct comparison to prior research using more conventional measures. On average, only two to three emotions were selected per entry, which leads to an overdispersion of zeros across emotion items and further complicates analysis. Importantly, the fact that certain items are not reported simultaneously does not imply that the emotions they represent belong to entirely separate constructs. For example, selecting “relaxed” instead of “calm” does not necessarily indicate a shift in emotional state—both still fall under the broader category of serenity. Nevertheless, the small overlap between individuals also means that we cannot study the full set of emotions, or even a subset of emotions, across the sample, which limits certain types of analyses. Together, these psychometric and design-related limitations highlight that while the DMA is highly valuable for capturing dynamic, person-specific emotion data, its factor structure, reliability, and validity are currently insufficiently established for traditional factor-based analyses. These considerations should be carefully taken into account when interpreting results, while also recognizing that they may reflect the unique advantages and constraints of the DMA’s design. Nevertheless, previous studies by Hoemann (2024) have also shown that freely reported emotions showed little overlap with features in commonly used affective scales. This mismatch underscores a broader concern: that standardized tools may underrepresent the true diversity of emotional experiences in daily life. While the DMA presents new challenges for data aggregation and traditional analysis, its flexible format opens promising avenues for investigating how emotions naturally emerge, co-occur, and are labeled across individuals. Future research could benefit from employing more dynamic and individualized analytical approaches, such as person-specific factor analysis, emotion network modeling, or dynamic time warping to compare emotional trajectories. Additionally, methods like hierarchical clustering or latent profile analysis could be used to group individuals with similar emotion use patterns, allowing researchers to study emotion dynamics at both individual and subgroup levels. Integrating qualitative methods (e.g., emotion diaries or cognitive interviews) may also provide insight into the meaning participants assign to different emotion terms, improving the interpretability of item selection in hybrid designs like the DMA. Importantly, these person-centered approaches should be complemented by rigorous psychometric validation steps, such as exploratory factor analysis (EFA), cross-validation in diverse samples, or comparative analyses with validated instruments. Combining dynamic, individualized methods with careful validation will help ensure that findings are both innovative and interpretable, while maintaining methodological rigor. The flexibility of such person-centered approaches opens exciting new avenues for emotion research—moving beyond rigid categorizations to embrace the complexity and individuality of everyday emotional life.

Another limitation is the study population. Even though the higher education sample was chosen on purpose, it led to a rather homogenous sample. The majority of the sample fell into the young age category and two-third consists of bachelor students of the education programs psychology and pedagogy of which around 80% was female. However, participation in this study was not limited to these faculties. We distributed flyers across different faculties of the VU Amsterdam and across the VU campus specifically targeting the male, non-binary or other identifying students. Even though this increased the number of non-cis-gender females to around a third of the study population it remains unbalanced imposing difficulties on studying gender effects. Such an unequal gender division of over 80% females is consistent with other studies on mood with EMA and wearables, such as the WARN-D study (Siepe et al., 2024), or addressed by a panel study of Stone and colleagues (2023) that showed that females were 30–35% more likely to participate than males.

Specifically, the large proportion of female participants may have influenced the emotional dynamics observed in this study. For instance, research has shown that females are more likely to experience heightened emotional sensitivity during stressful periods, for example during the covid19 pandemic (García-Fernández et al., 2021; Maslakçı & Sürücü, 2024; Peyer et al., 2024; Prowse et al., 2021). Thus, the findings related to emotional trajectories and recovery after the exam may reflect gendered patterns that are not universally applicable. The homogeneity of the sample in terms of academic discipline and educational level also limit the generalizability of the findings. For example, individuals growing up in adverse socioeconomic condition are at increased risk of childhood and life course stressors which shapes their acute stress response (Crielaard et al., 2021). In light of these limitations, future research should validate the DMA across diverse samples that better represents different genders, academic backgrounds, and age groups to determine whether the patterns observed in this study hold across broader and more varied populations.

## Conclusion

The aim of this study was to explore the feasibility of new methods for measuring the complexity of everyday emotional experiences. The DMA survey emerges as a promising and innovative tool for capturing emotional dynamics in daily life. It allows for the assessment of a wide range of emotions without significantly increasing response time or decreasing participant adherence, making it a practical option for large-scale experience sampling research. Despite some challenges in applying traditional statistical analyses due to variability in emotion reporting, the DMA offers distinct advantages, particularly for researchers interested in emotional granularity. By capturing subtle variations in how individuals report their emotions, it provides a unique opportunity to examine the structure of emotional experiences with a high degree of specificity. Furthermore, the DMA has the potential to uncover individualized patterns of emotional change in response to real-life stressors, helping to identify which individuals demonstrate resilience and which may exhibit heightened emotional reactivity to stress. Additionally, it offers valuable insights into between-person differences in emotion dynamics, contributing to a more personalized understanding of emotional regulation processes. However, the generalizability of our findings is limited by the sample used in this study. To rigorously evaluate the feasibility and applicability of the DMA, future research should collect data across diverse populations, including different age groups and social economic status. Moreover, refinements may be needed in the selection of items included in the DMA, as well as in the methodological approach used to analyze the data, given the overrepresentation of zero responses across multiple emotion items.

Taken together, our findings suggest that the DMA provides a foundation for future research aimed at capturing the nuanced nature of emotional experiences in everyday life. As a methodological innovation, it provides researchers with the ability to explore individual differences in emotional responses and examine their implications for psychological well-being and intervention strategies. By enabling a more fine-grained analysis of emotional dynamics, the DMA has the potential to become a valuable addition to the toolkit for studying emotions in real-world contexts.

## Supplementary Information

Below is the link to the electronic supplementary material.

Supplementary File 1 (PDF 13.0 MB)

Supplementary File 2 (DOCX 238 KB)

## Data Availability

All data and documentation are freely available on the projects Open Science Framework page: https://osf.io/rd9pm/.
