# Supplementary figures and images for: Tracking everyday emotional experiences in university students with the distinct mood assessment questionnaire
Source: Curr Psychol. 2026 Feb 27;45(6):605. doi: 10.1007/s12144-025-08991-6 (PMC12948869; doi:10.1007/s12144-025-08991-6)

**Supplementary Figure 1.**


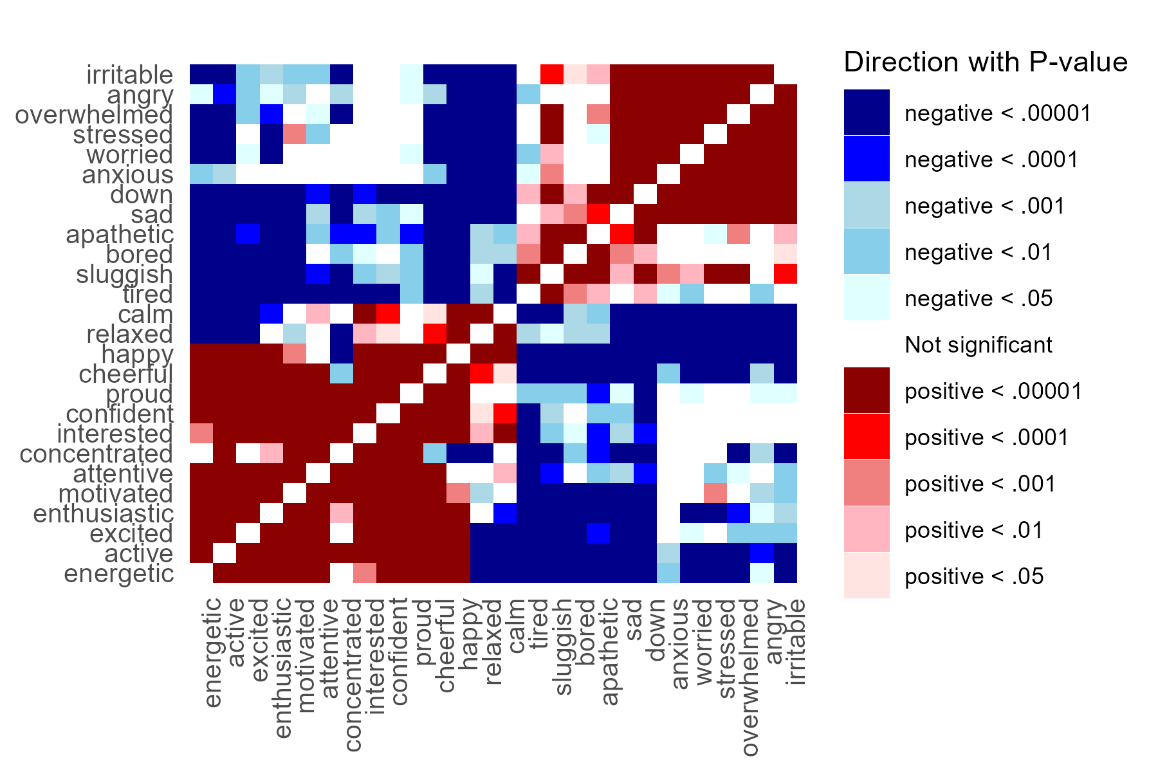
*Pairwise chi-square comparison of emotion items.*

Supplement: Supplementary file 2 — (DOCX 238 KB) [file 12144_2025_8991_MOESM2_ESM.docx]
