# Supplementary material for: Tracking everyday emotional experiences in university students with the distinct mood assessment questionnaire
Source: Curr Psychol. 2026 Feb 27;45(6):605. doi: 10.1007/s12144-025-08991-6 (PMC12948869; doi:10.1007/s12144-025-08991-6)
Supplement: Supplementary file 1 — (PDF 13.0 MB) [file 12144_2025_8991_MOESM1_ESM.pdf]

Appendix 1: Individual differences in change of emotions over time

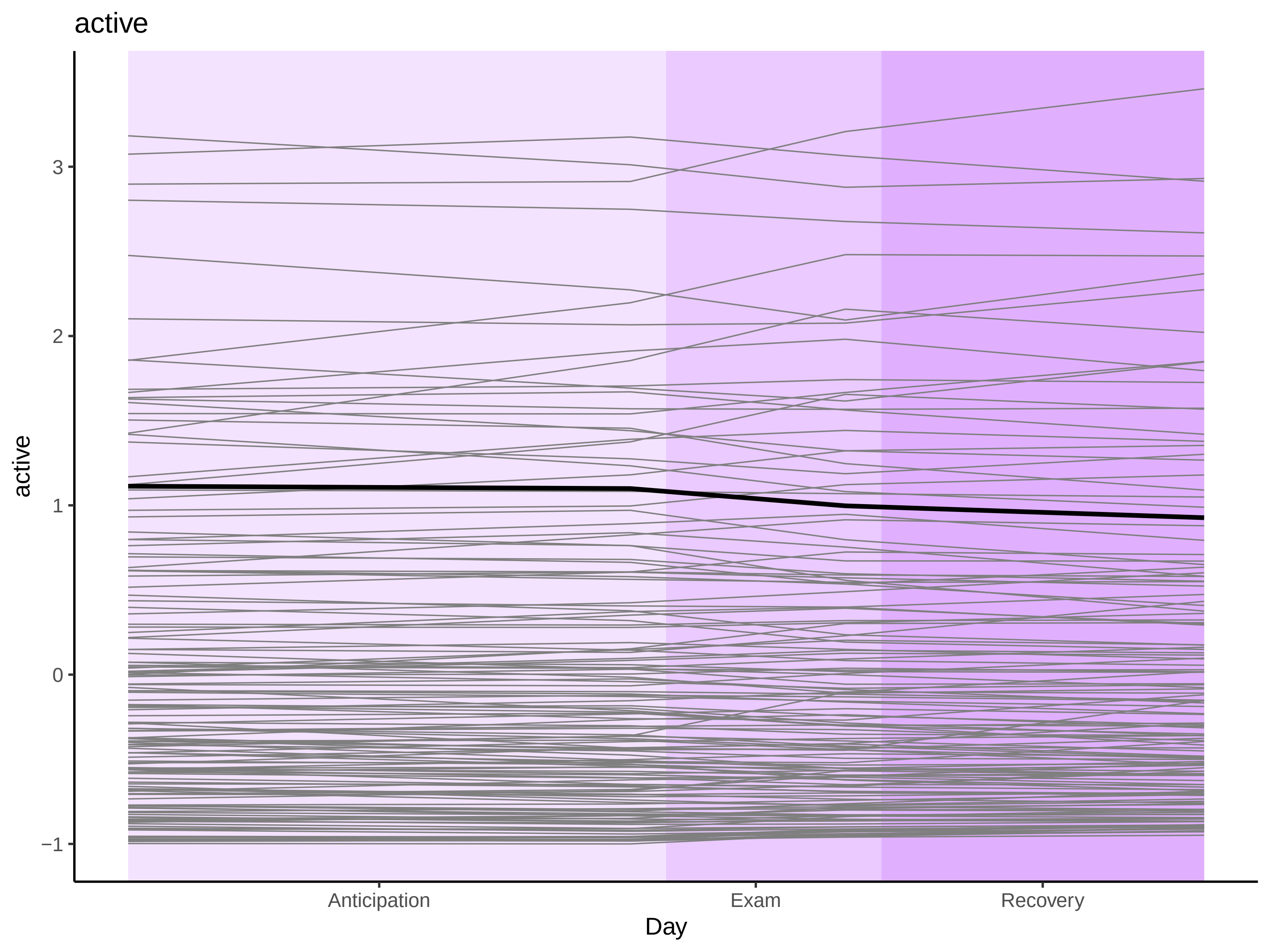
active
active
3
2
1
0
−1
Anticipation
Exam
Recovery
Day

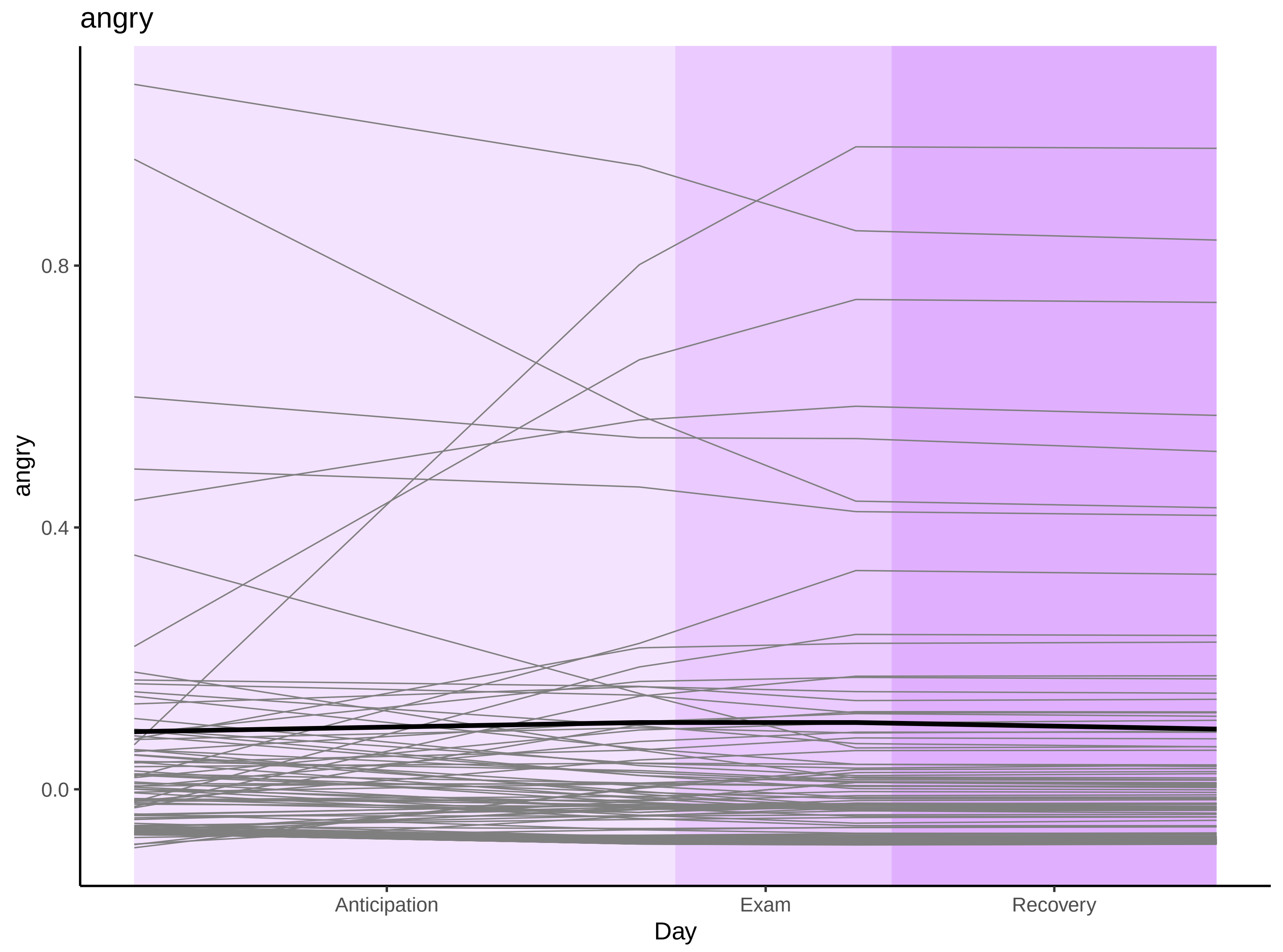

angry
angry
0.8
0.4
0.0
Anticipation
Exam
Recovery
Day

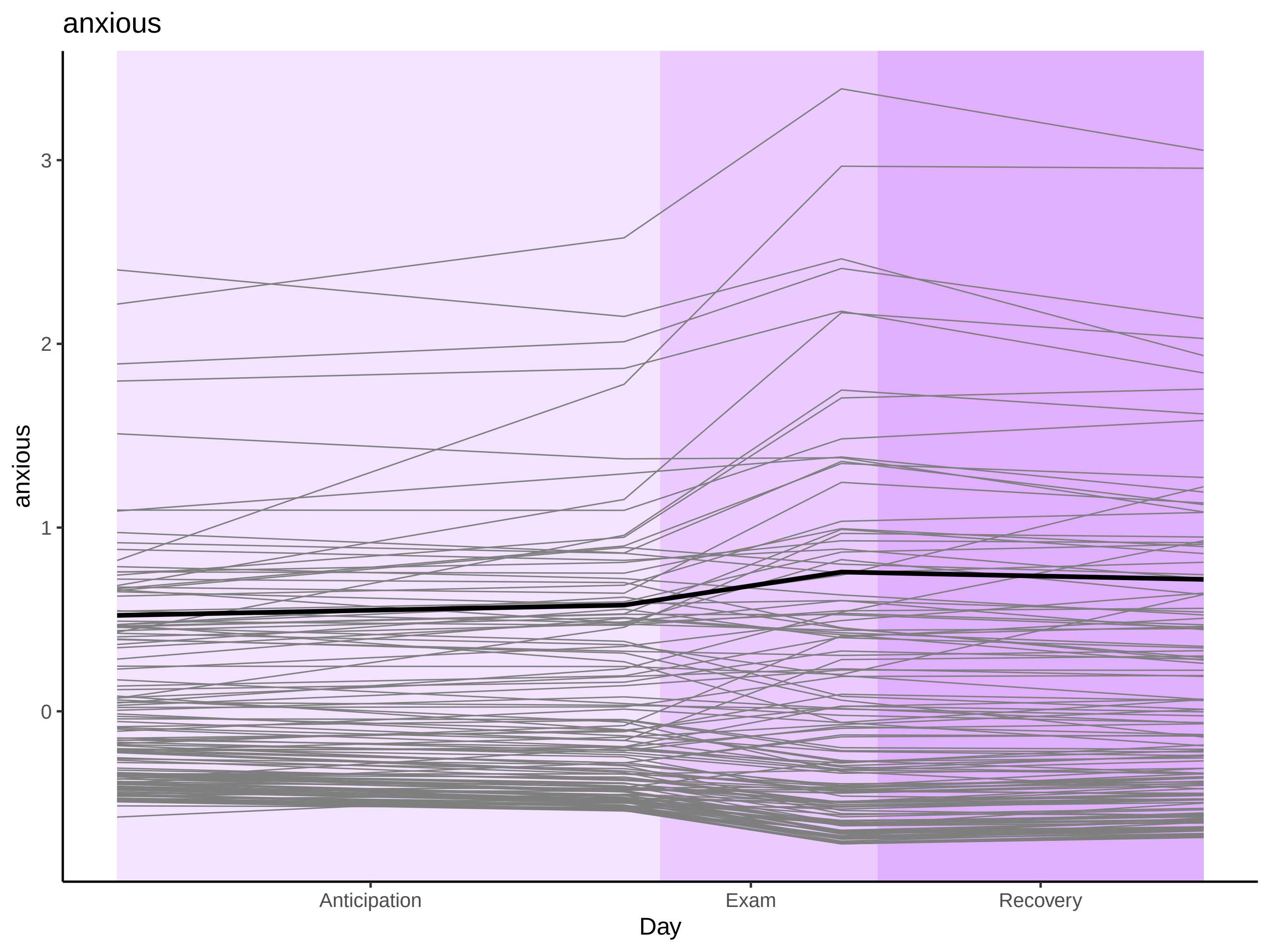

anxious
3
2
1
0
anxious
Anticipation
Exam
Recovery
Day

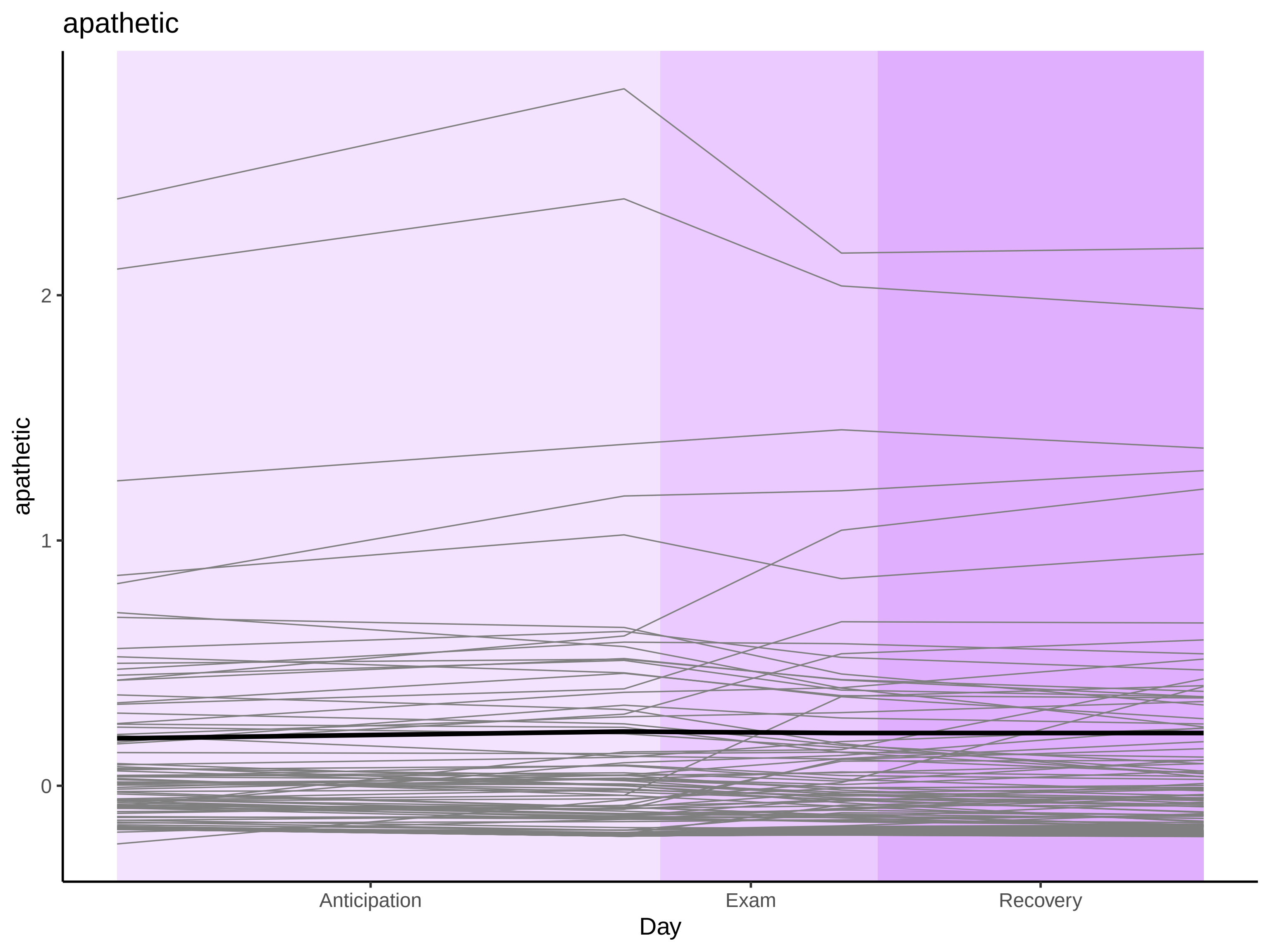

apathetic
apathetic
2
1
0
Anticipation
Exam
Recovery
Day

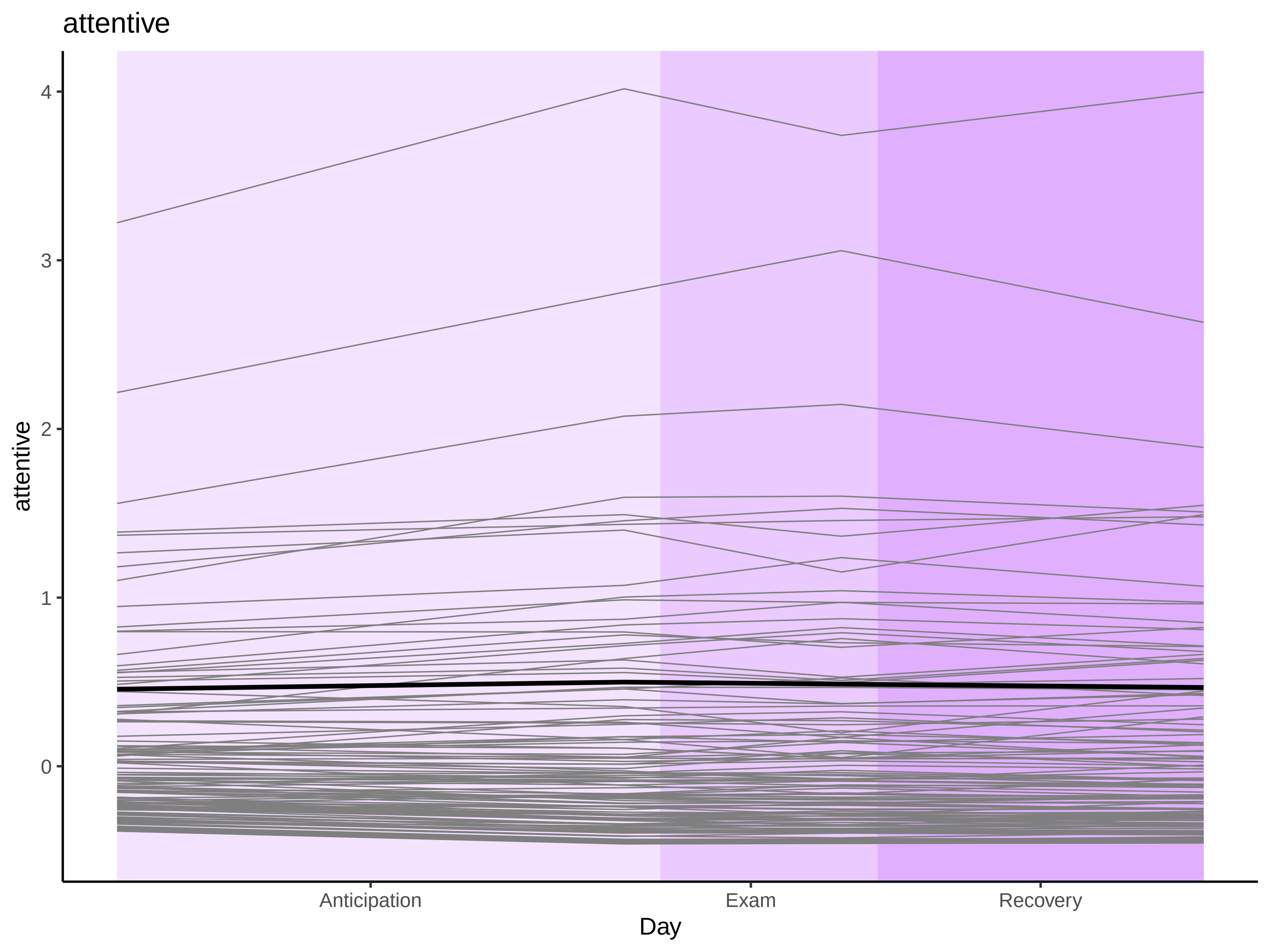

attentive
4
3
2
1
0
attentive
Anticipation
Exam
Recovery
Day

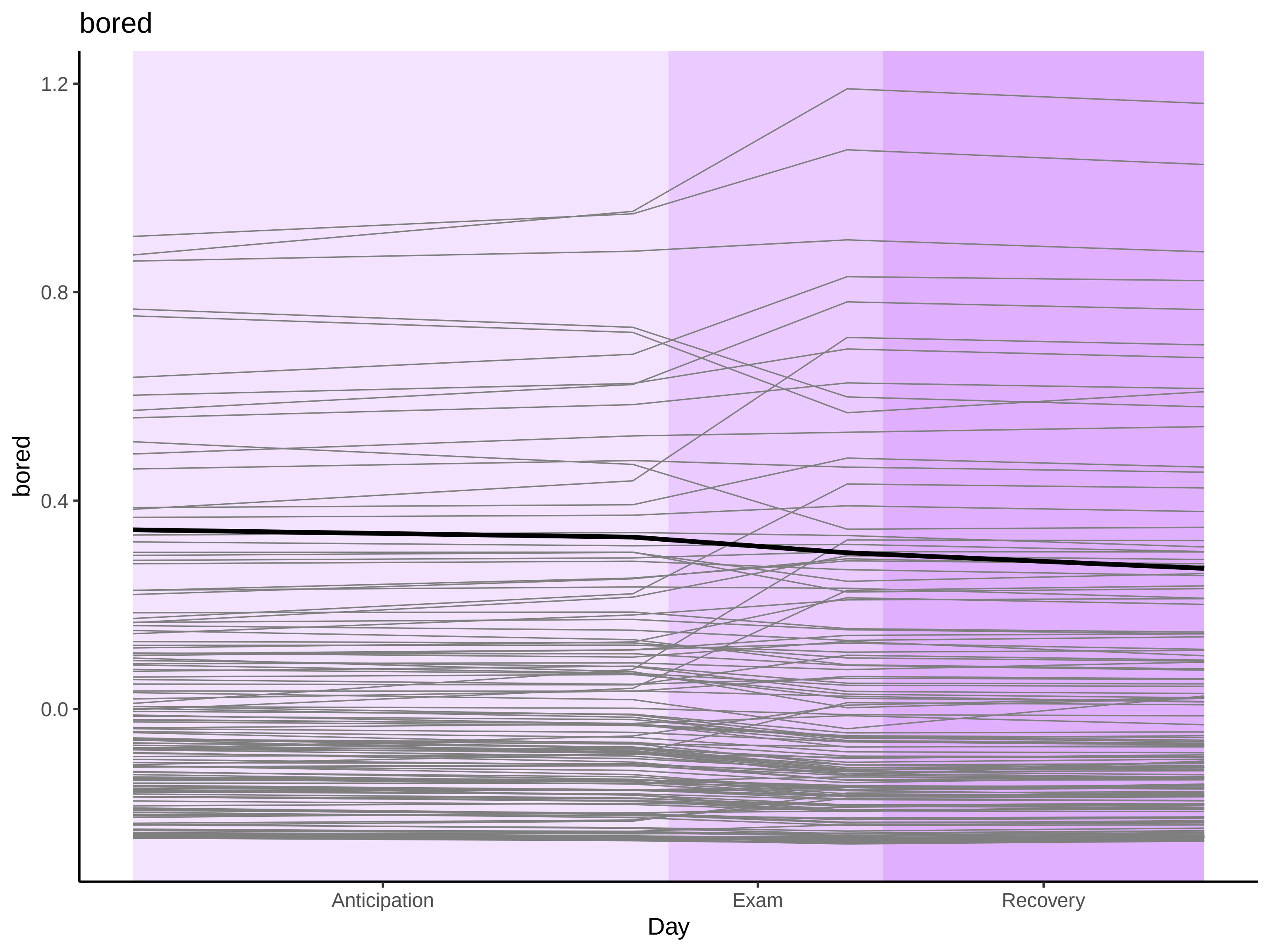
bored
1.2
0.8
0.4
0.0
bored
Anticipation
Exam
Recovery
Day

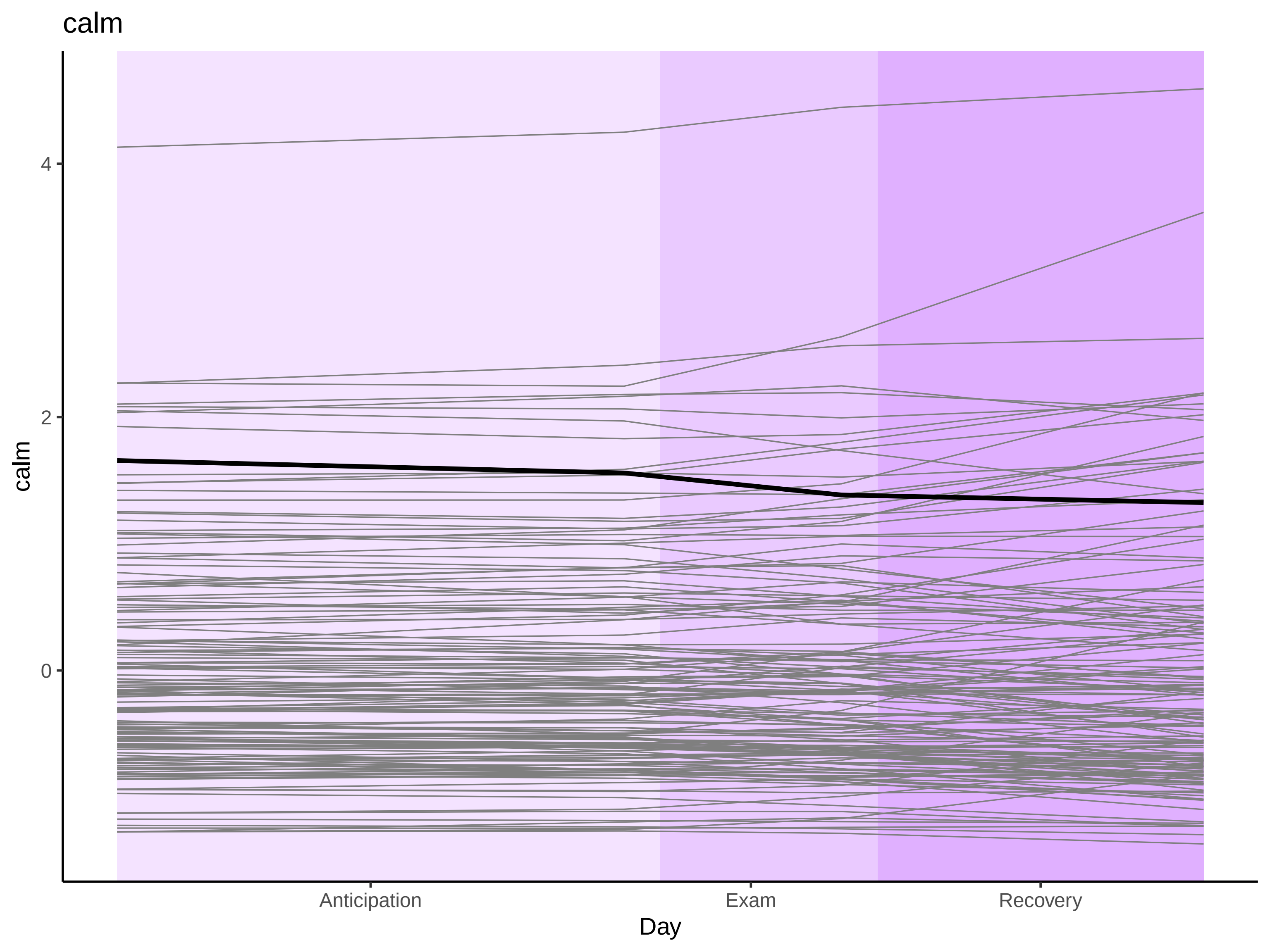

calm
4
2
0
calm
Anticipation
Exam
Recovery
Day

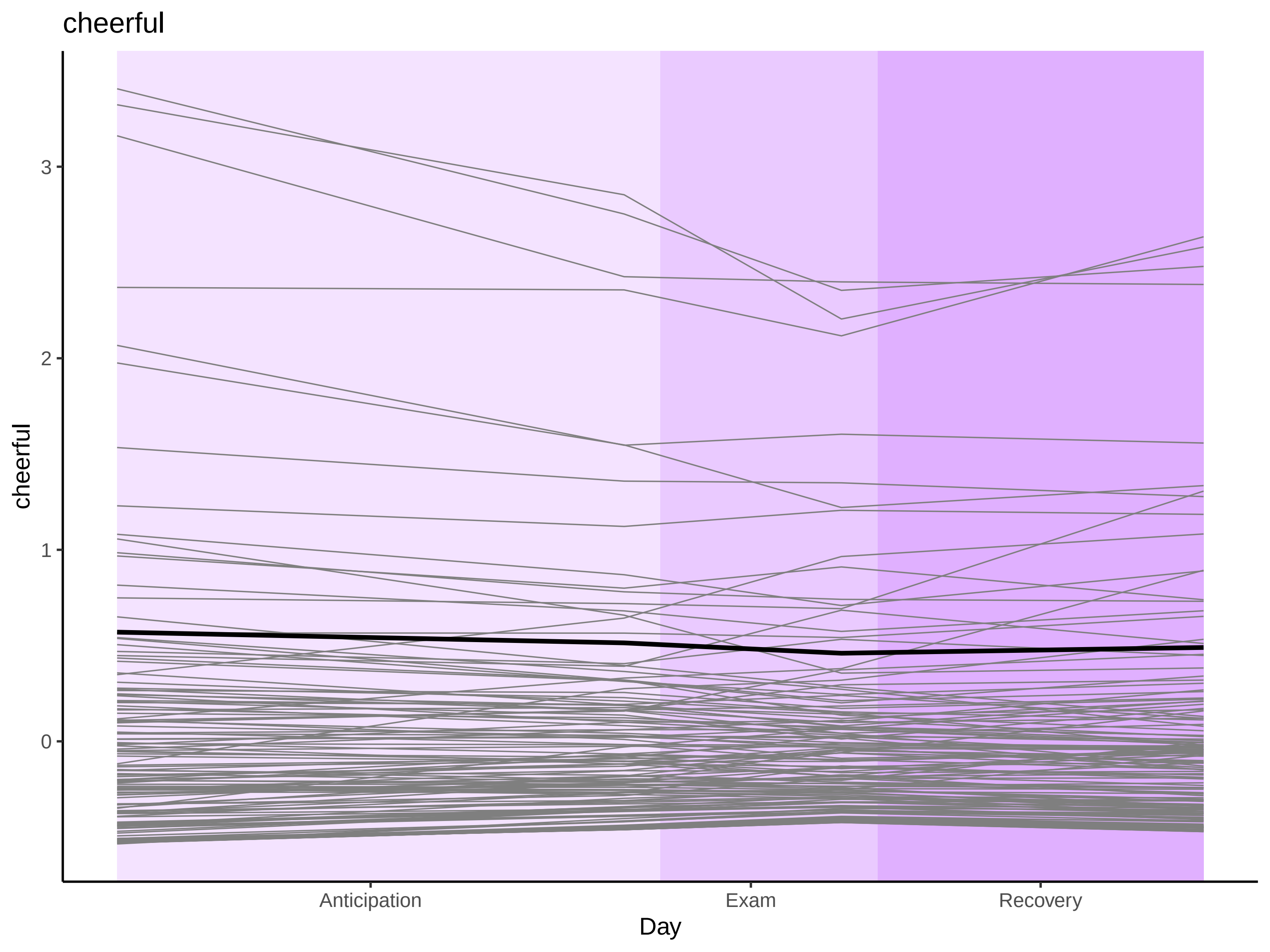
cheerful
3
2
1
0
cheerful
Anticipation
Exam
Recovery
Day

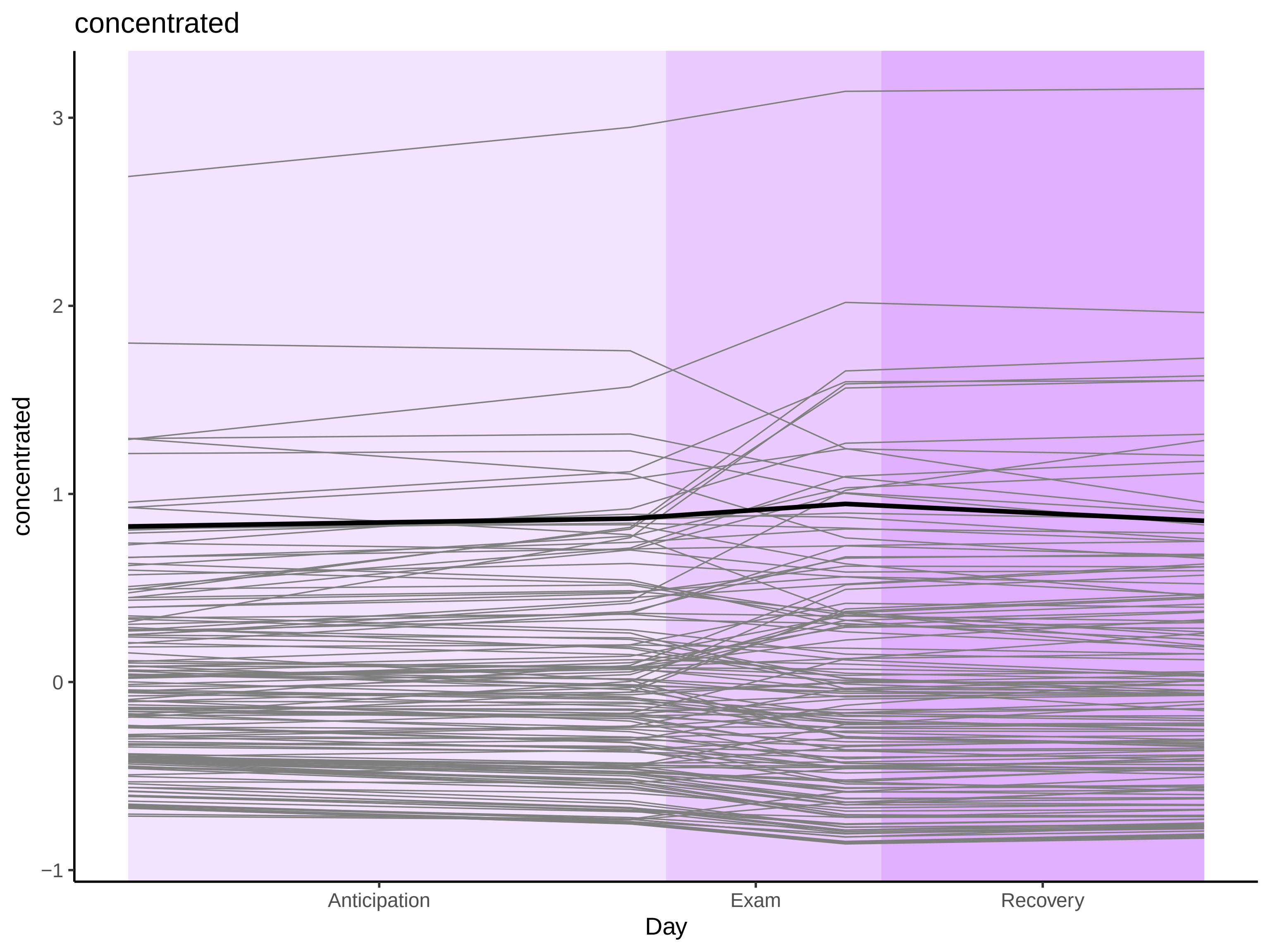

concentrated
3
2
1
0
−1
concentrated
Anticipation
Exam
Recovery
Day

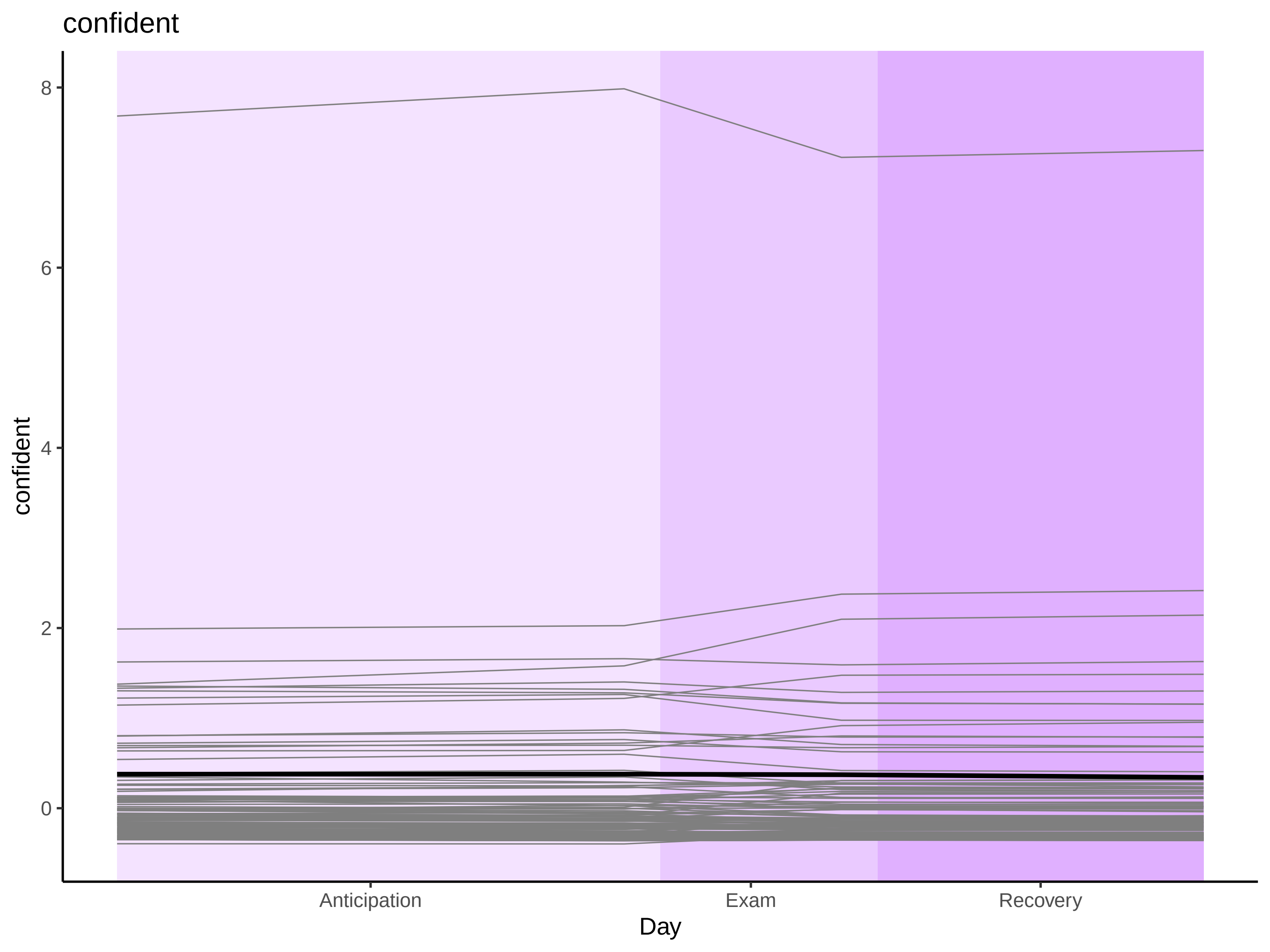
confident
8
6
4
2
0
confident
Anticipation
Exam
Recovery
Day

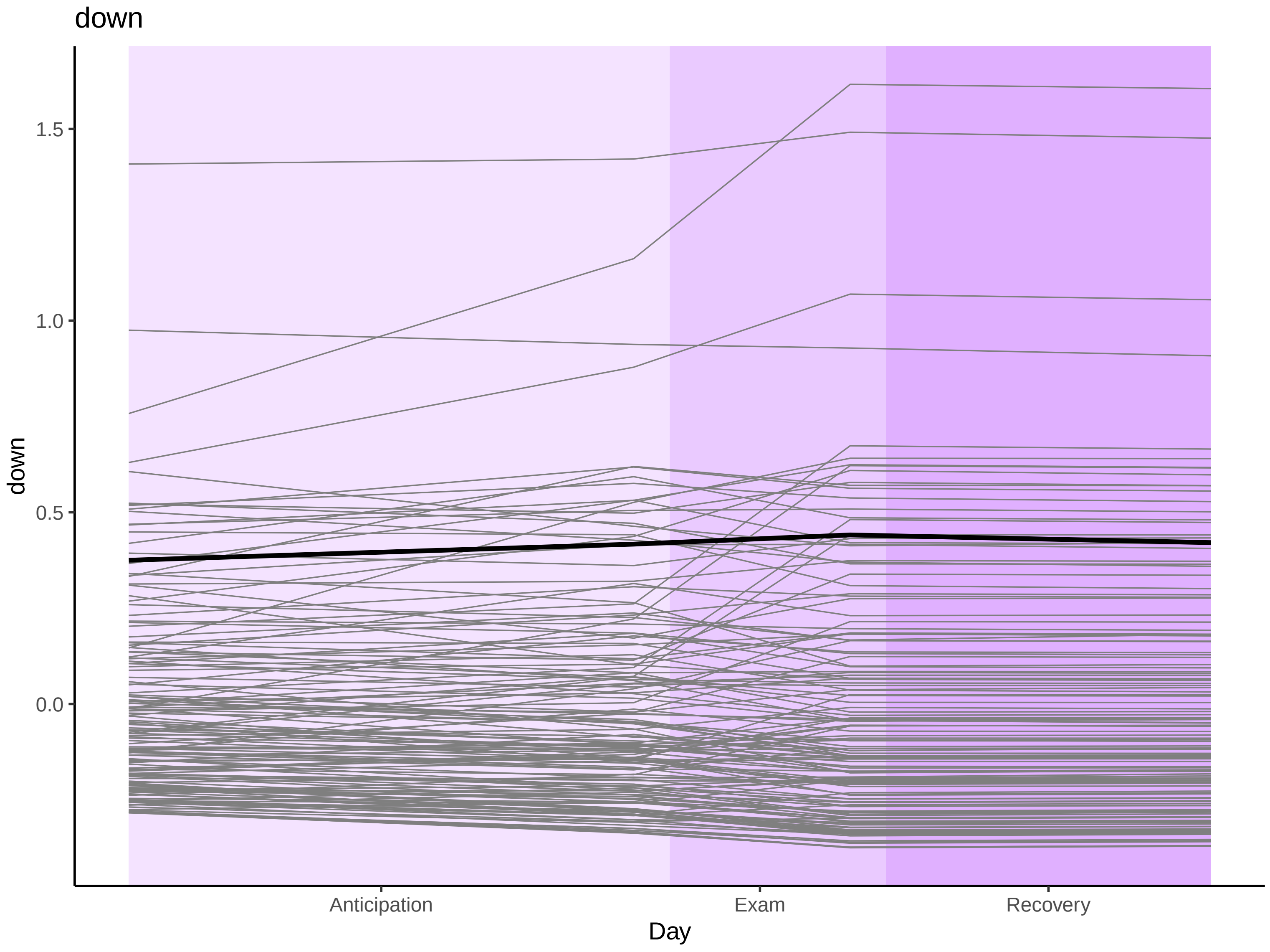
down
1.5
1.0
0.5
0.0
down
Anticipation
Exam
Recovery
Day

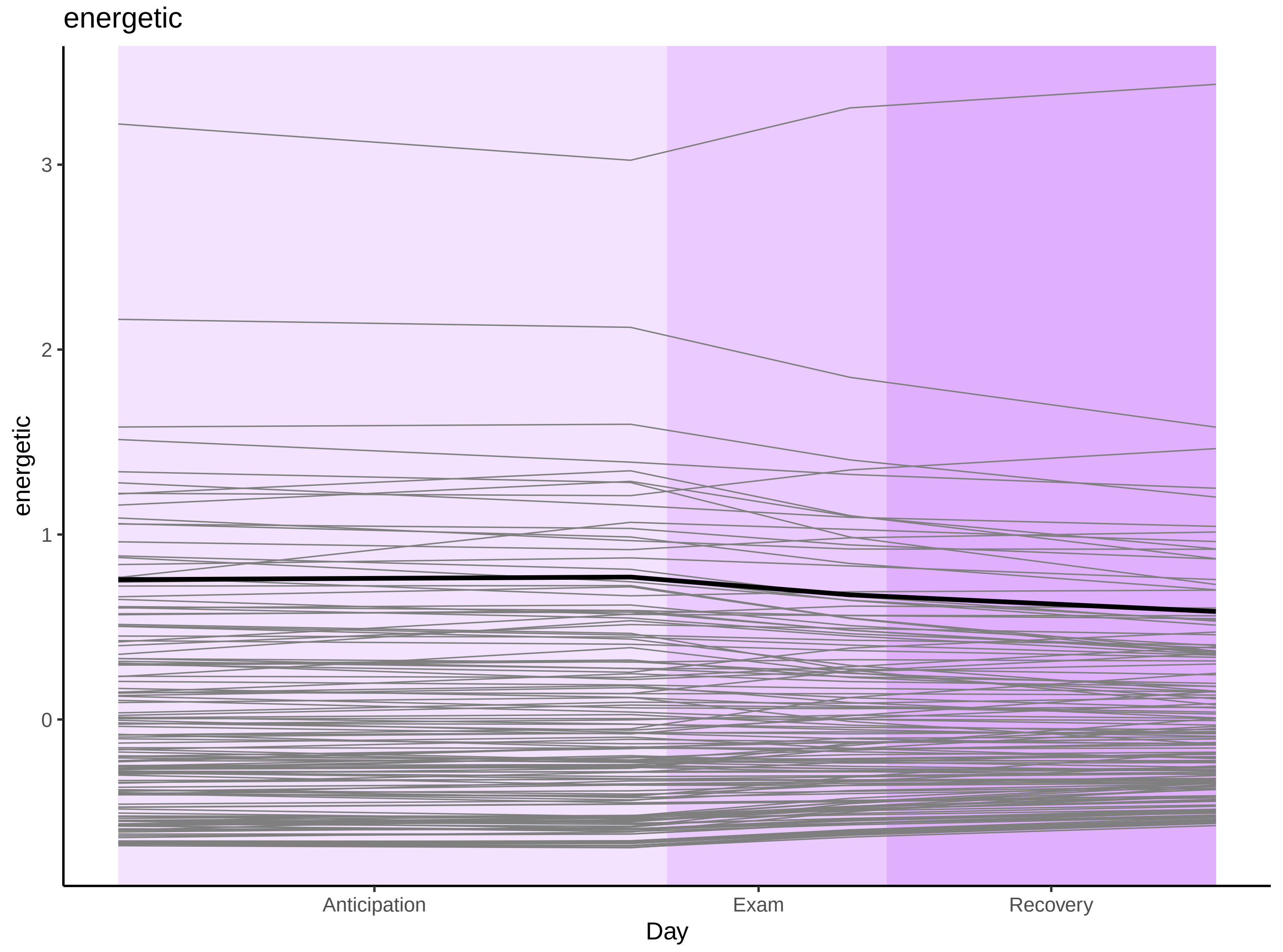
energetic
3
2
1
0
energetic
Anticipation
Exam
Recovery
Day

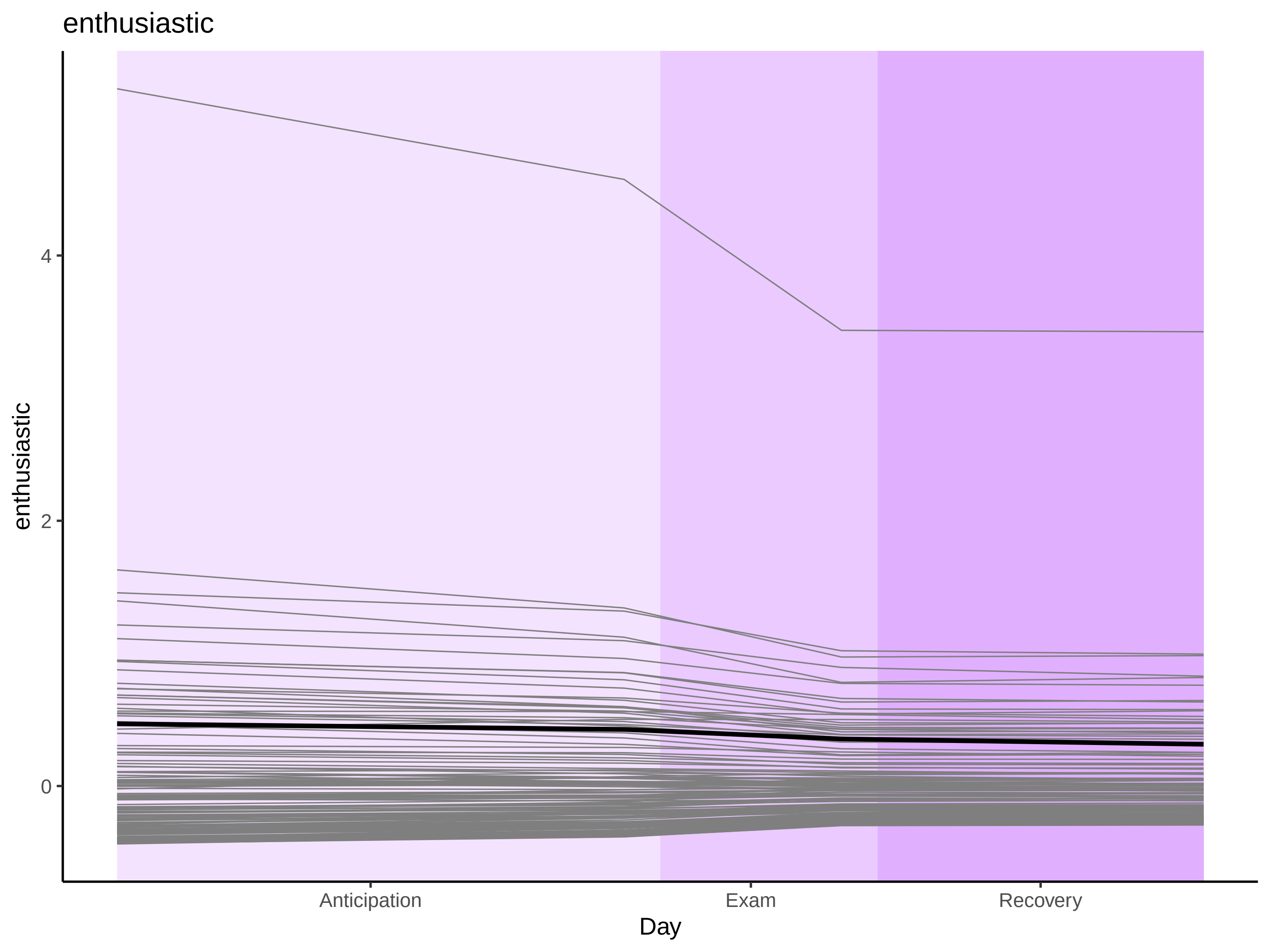
enthusiastic
enthusiastic
4
2
0
Anticipation
Exam
Recovery
Day

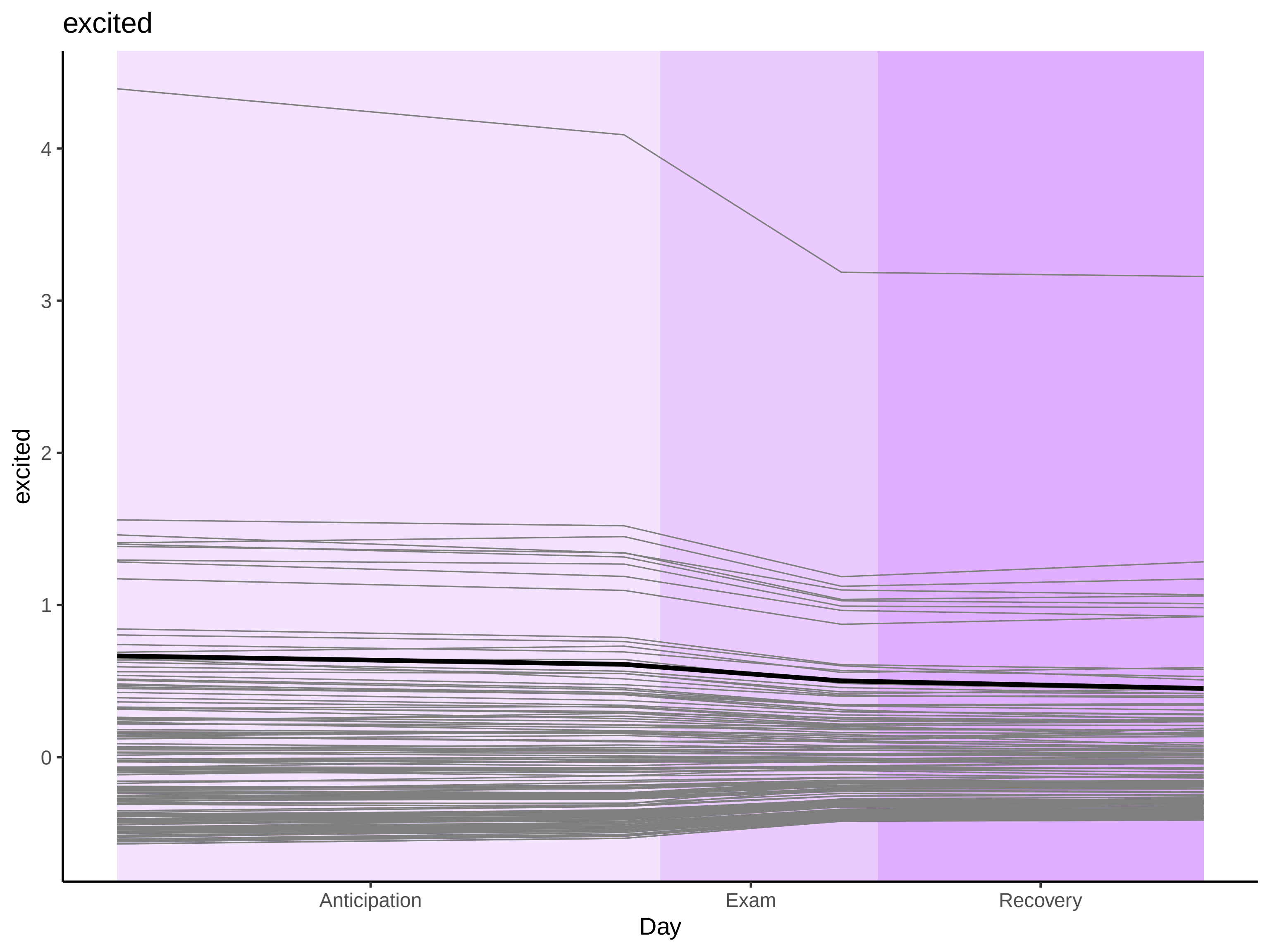
excited
excited
4
3
2
1
0
Anticipation
Exam
Recovery
Day

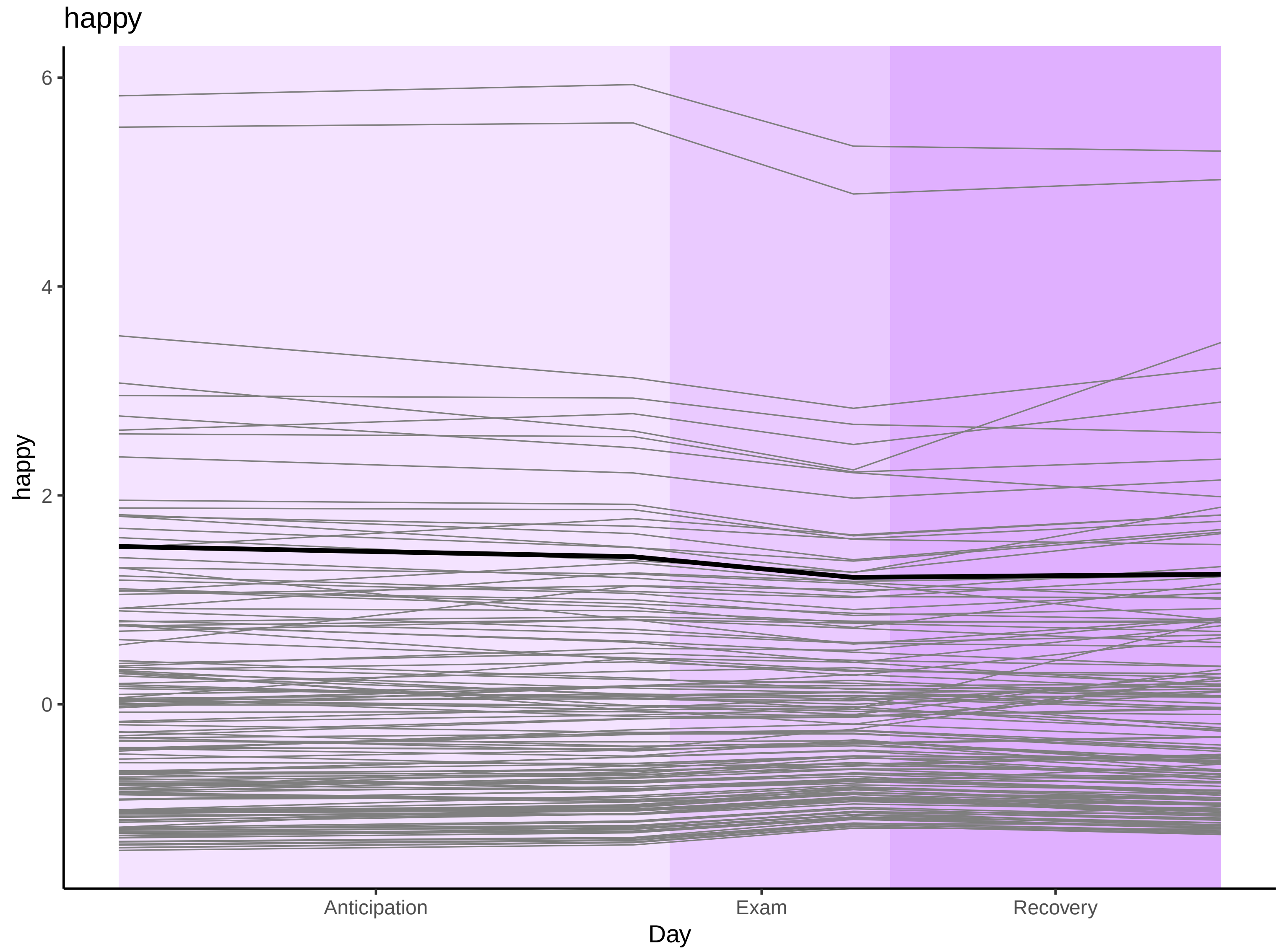
happy
6
4
2
0
happy
Anticipation
Exam
Recovery
Day

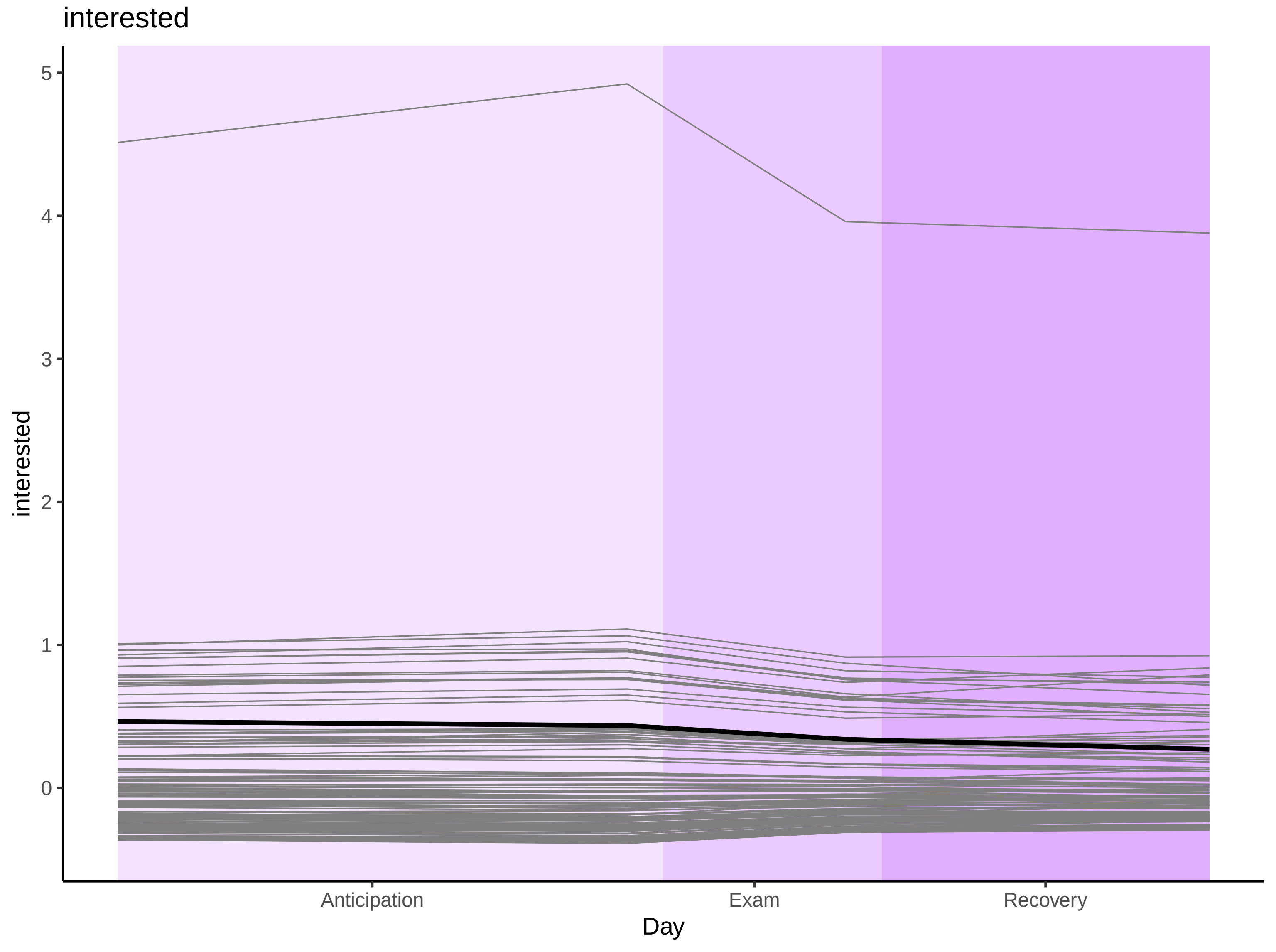
interested
5
4
3
2
1
0
interested
Anticipation
Exam
Recovery
Day

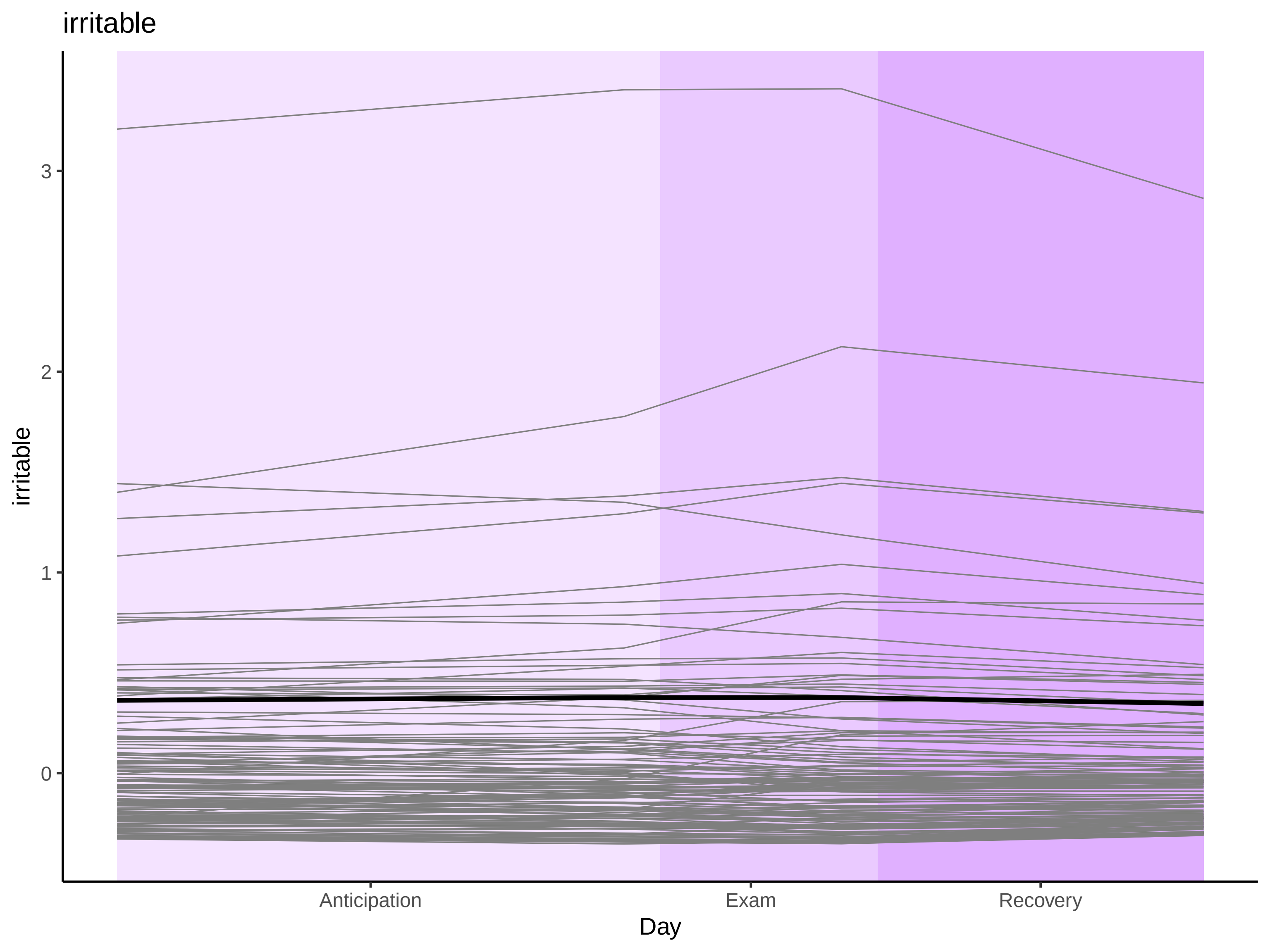
irritable
irritable
3
2
1
0
Anticipation
Exam
Recovery
Day

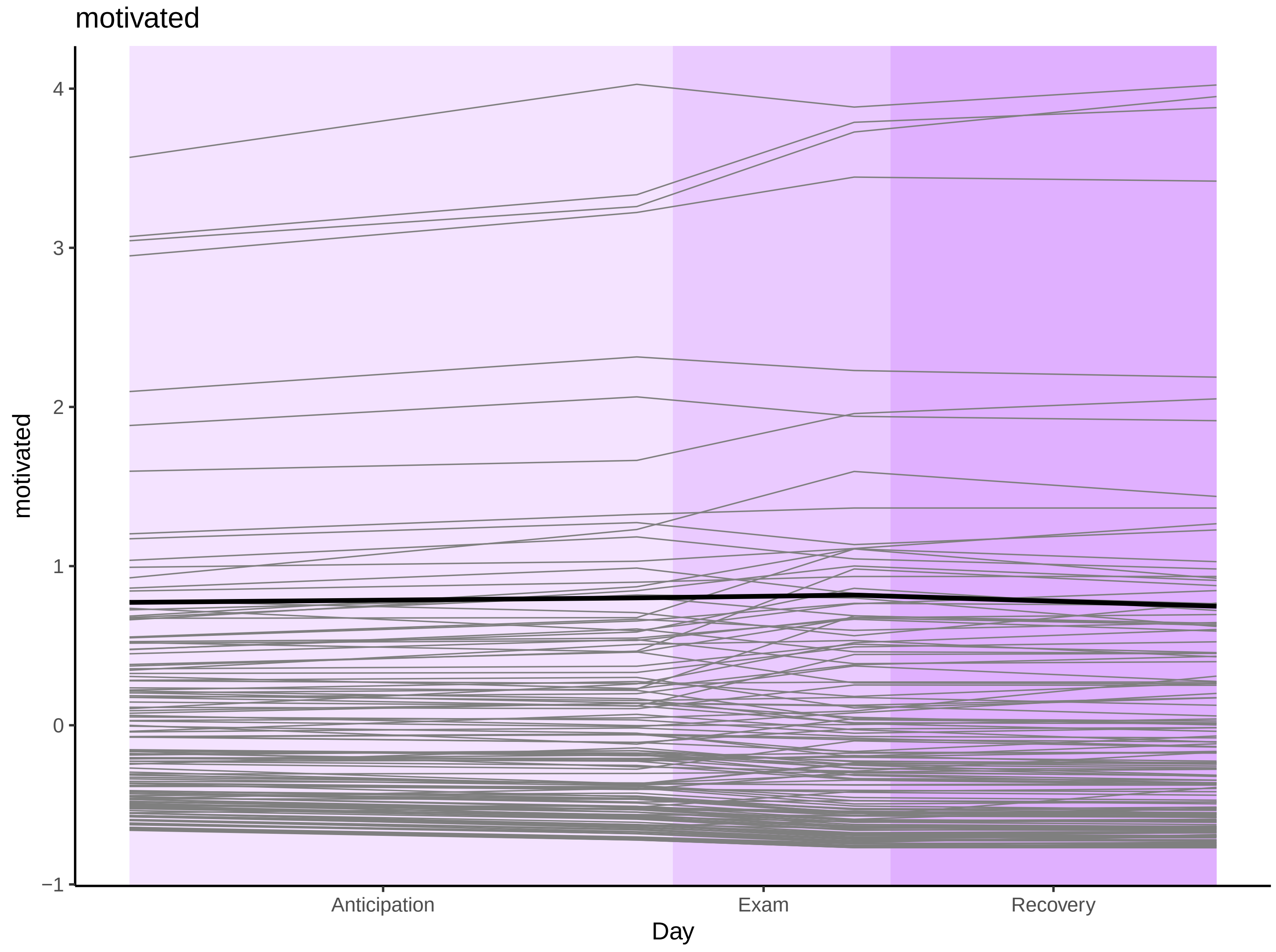
motivated
4
3
2
1
0
−1
motivated
Anticipation
Exam
Recovery
Day

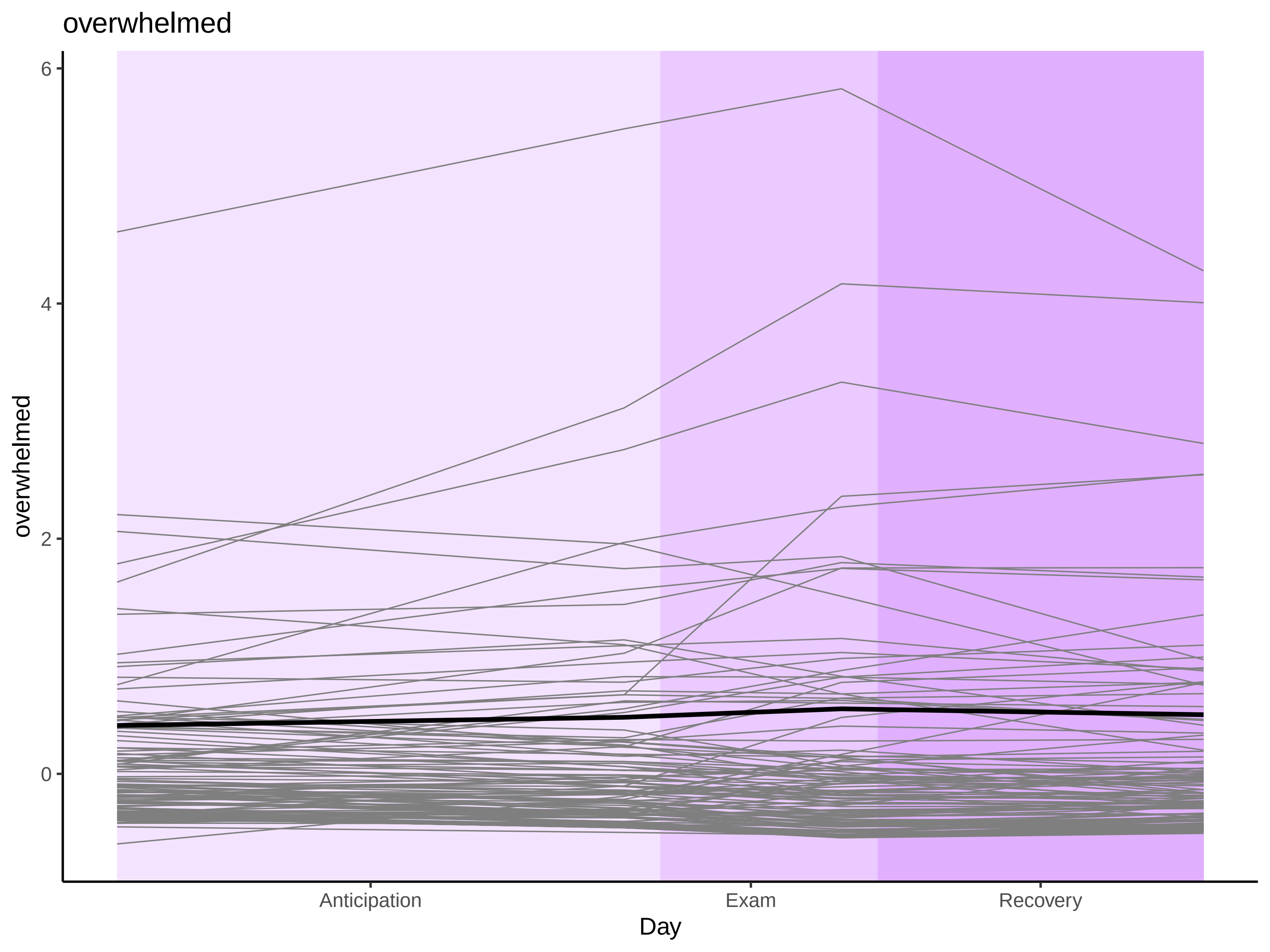
overwhelmed
6
4
2
0
overwhelmed
Anticipation
Exam
Recovery
Day

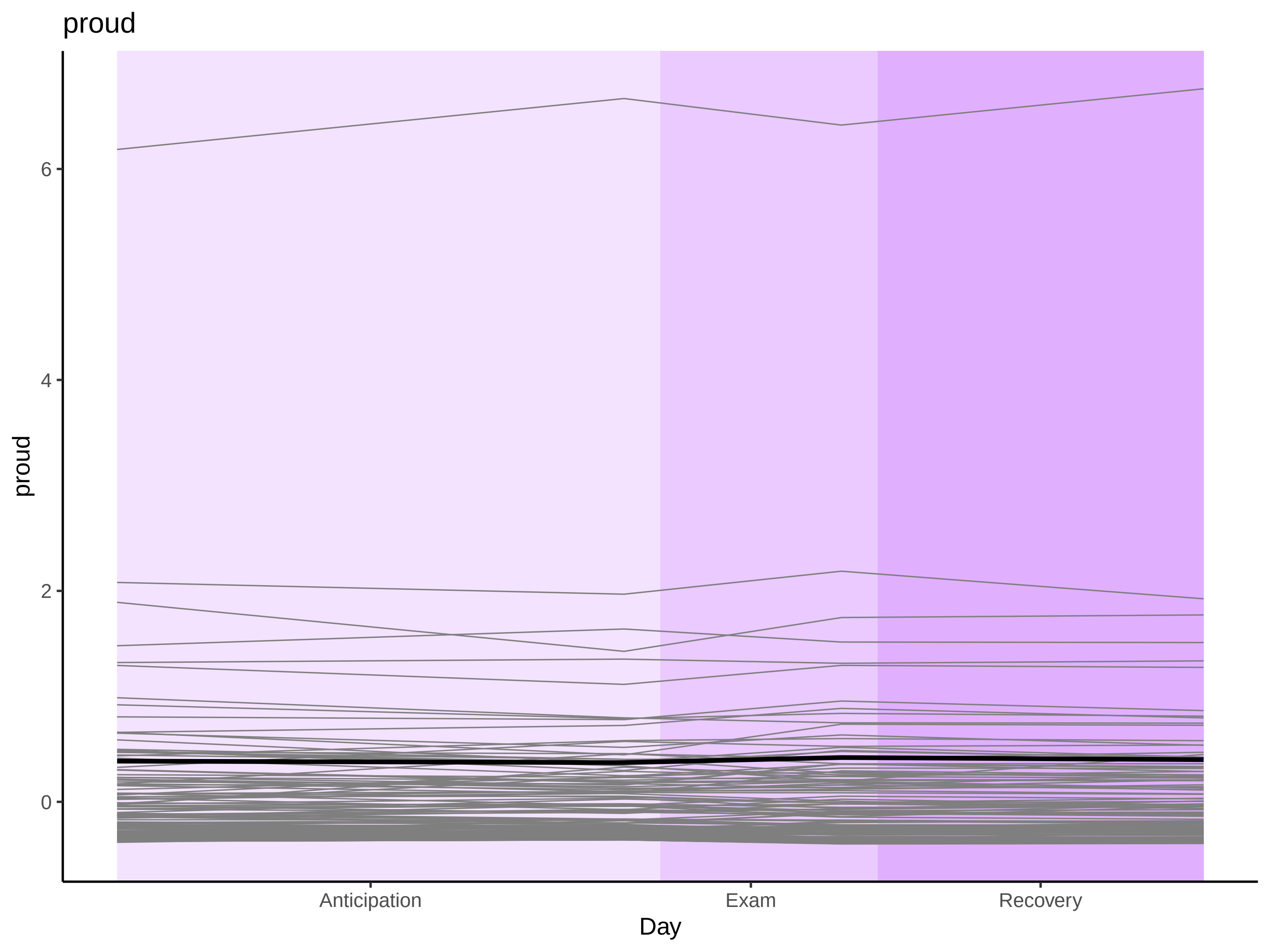
proud
6
4
2
0
proud
Anticipation
Exam
Recovery
Day

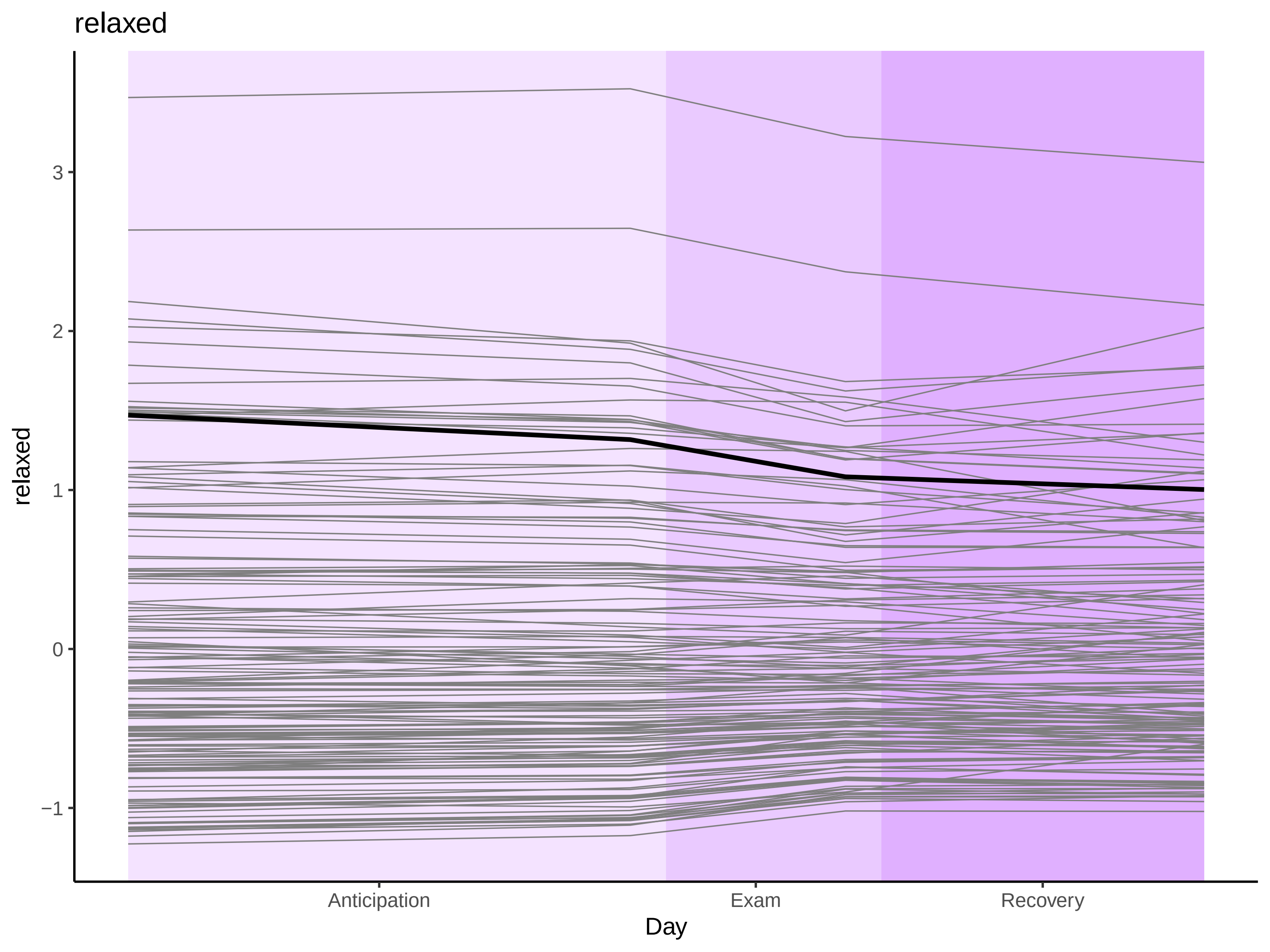
relaxed
3
2
1
0
−1
relaxed
Anticipation
Exam
Recovery
Day

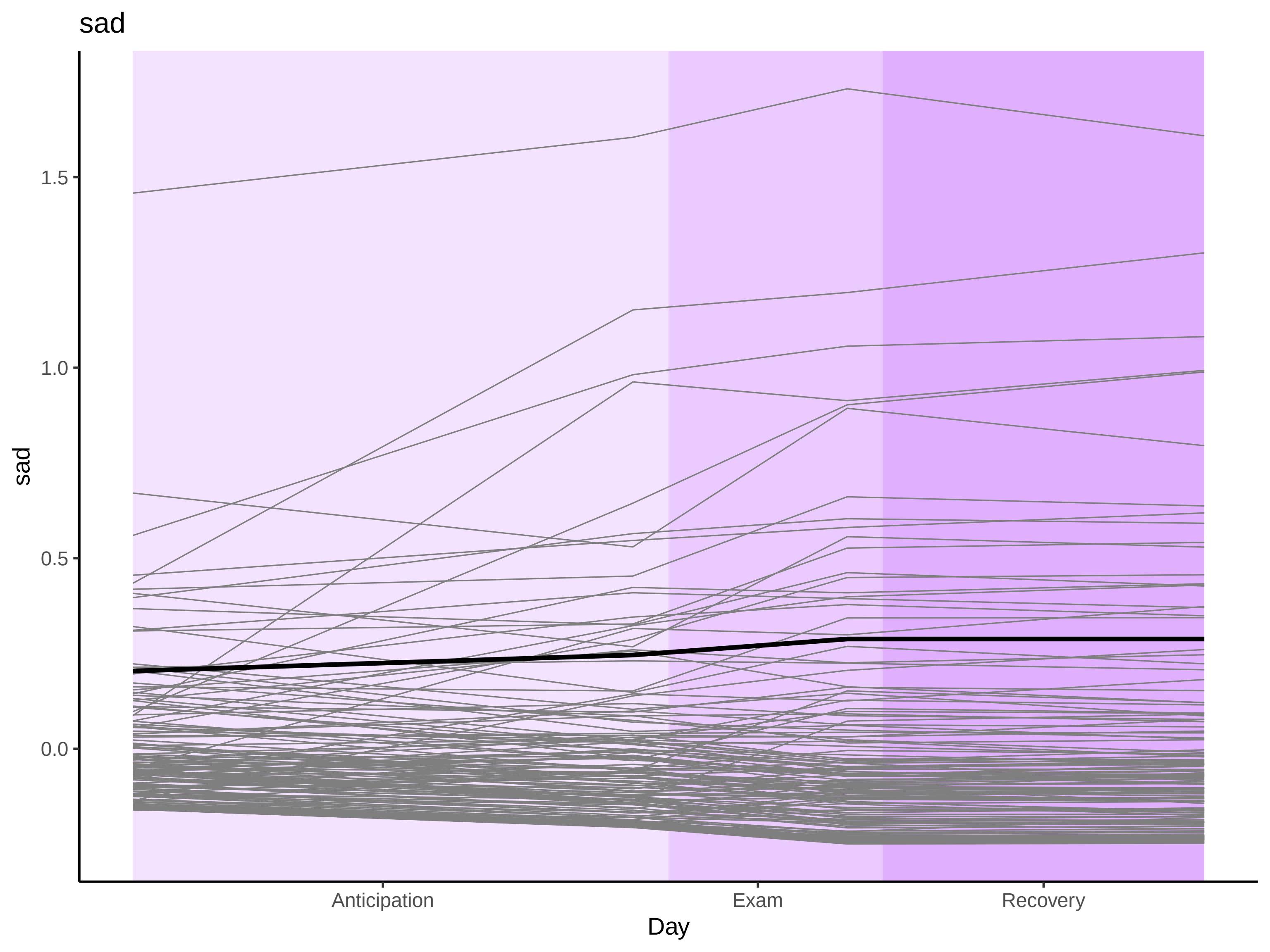
sad
1.5
1.0
0.5
0.0
sad
Anticipation
Exam
Recovery
Day

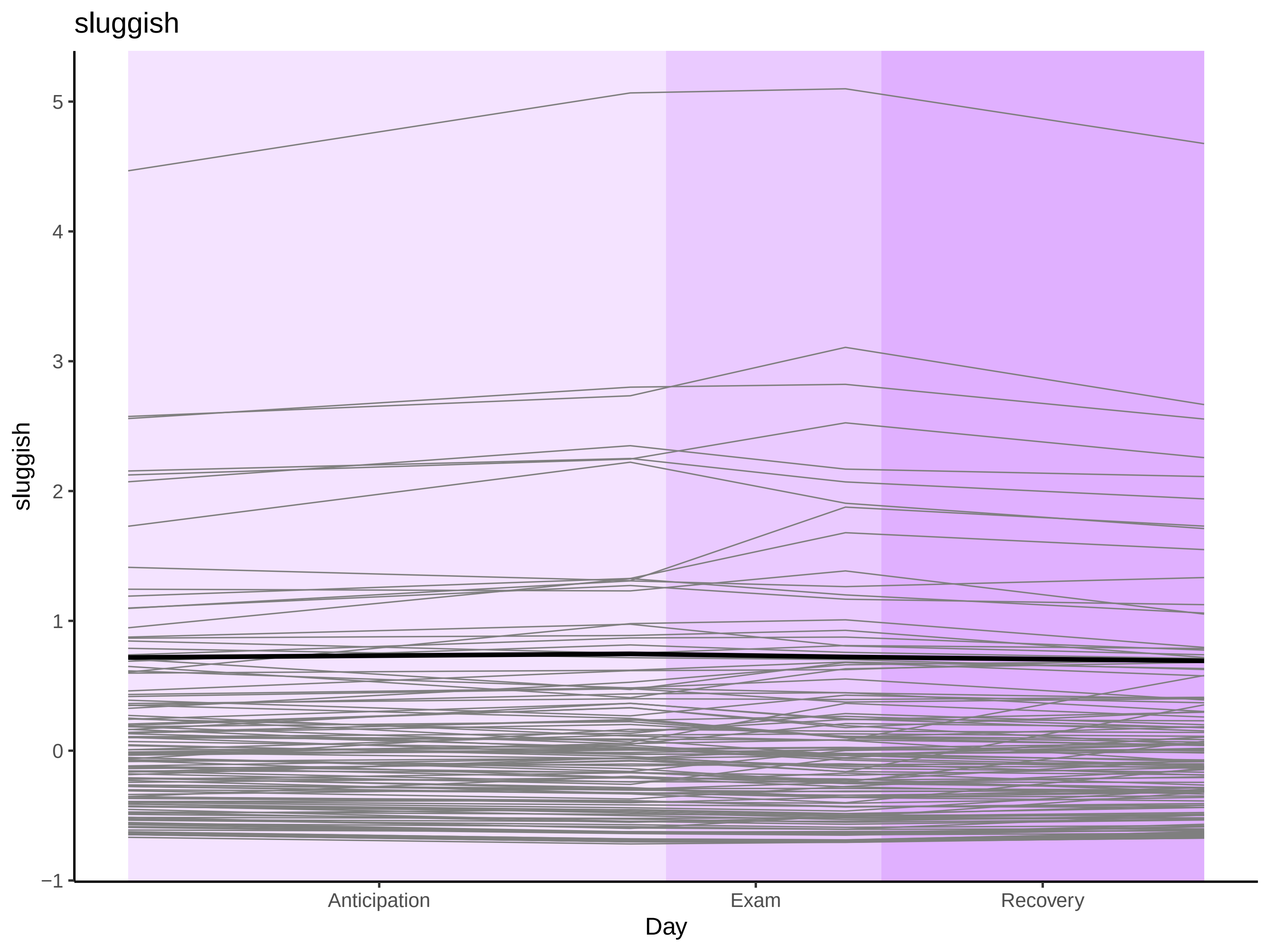
sluggish
sluggish
5
4
3
2
1
0
−1
Anticipation
Exam
Recovery
Day

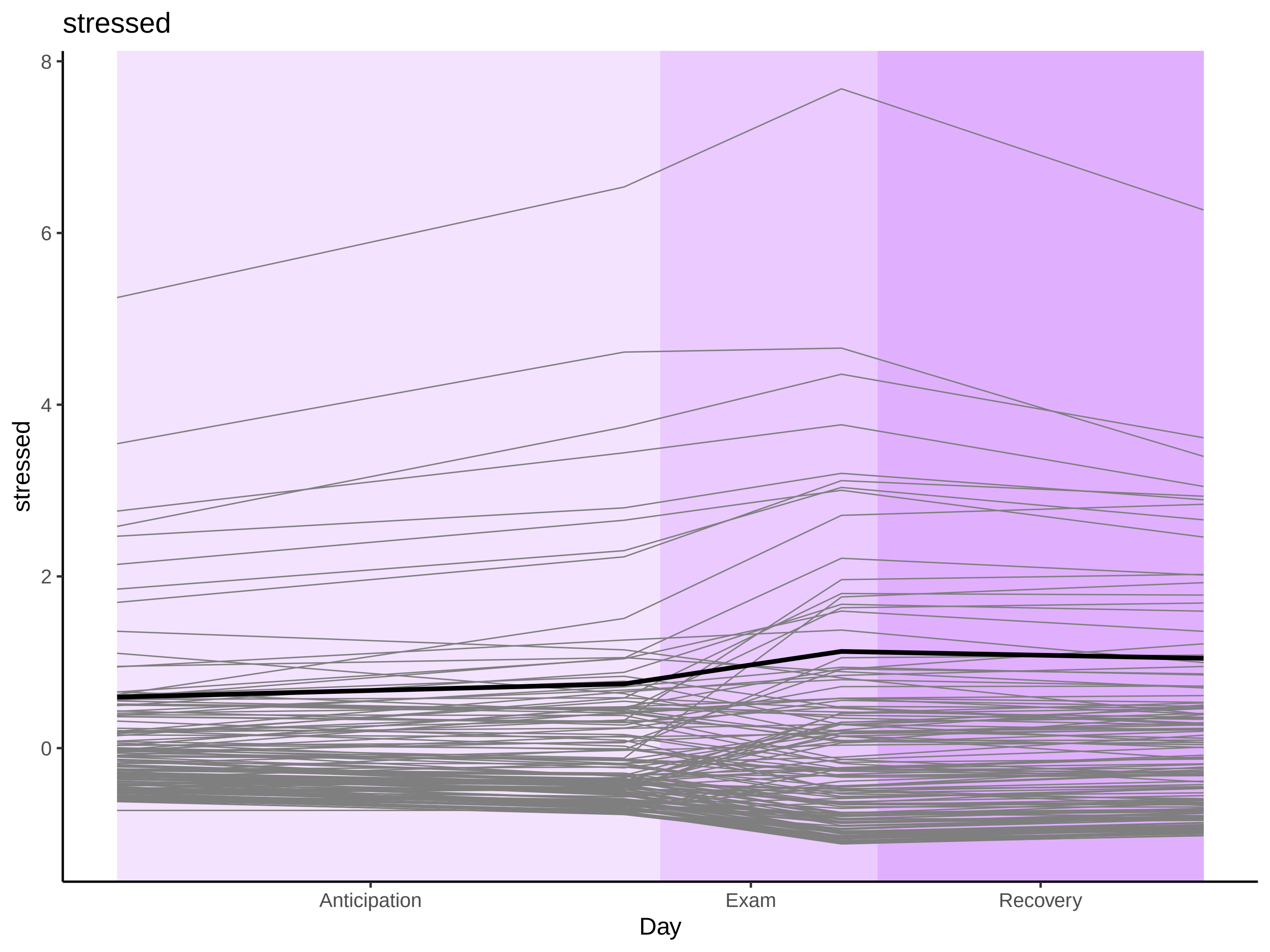
stressed
8
6
4
2
0
stressed
Anticipation
Exam
Recovery
Day

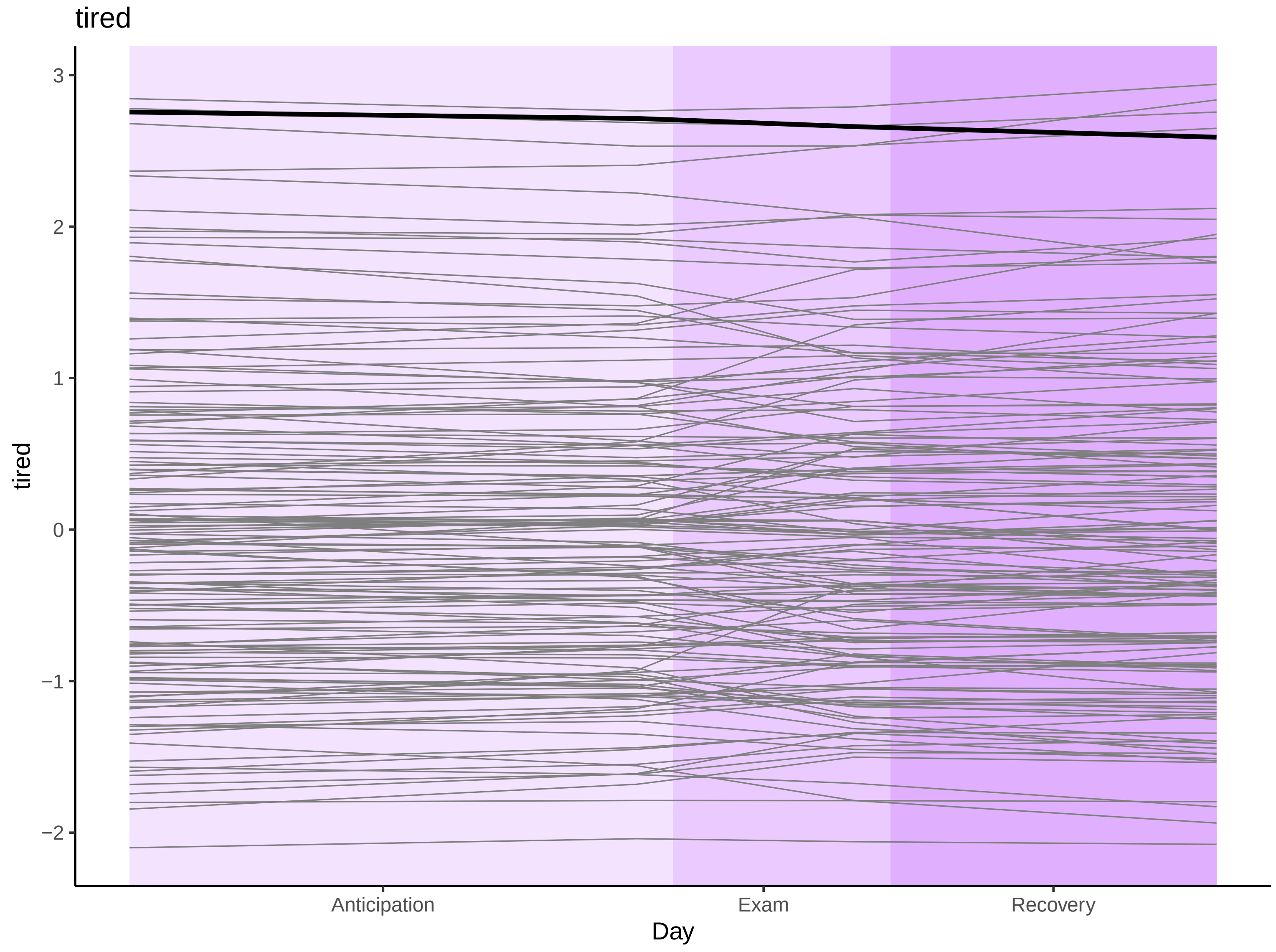
tired
3
2
1
0
−1
−2
tired
Anticipation
Exam
Recovery
Day

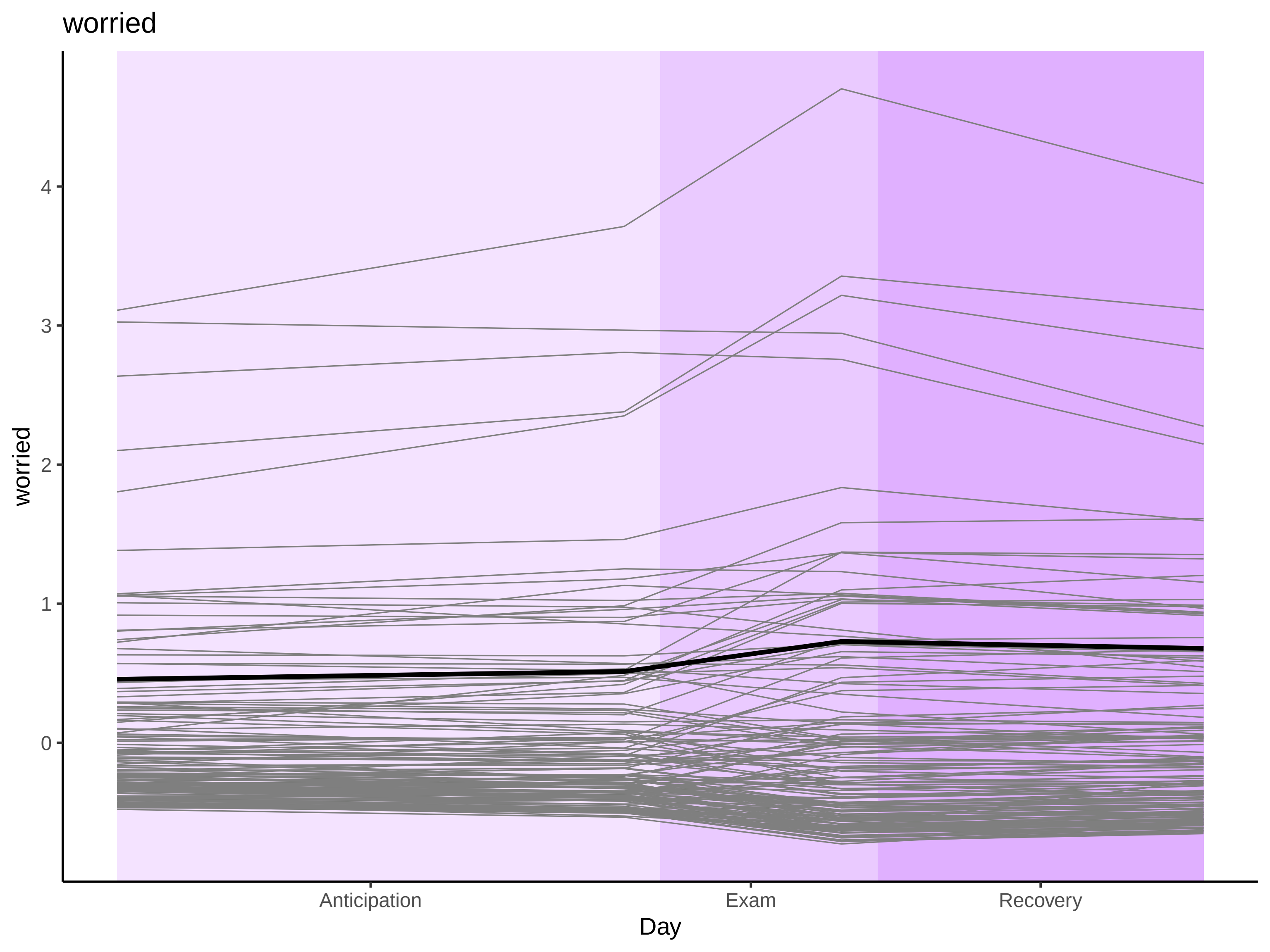

worried
4
3
2
1
0
worried
Anticipation
Exam
Recovery
Day
